# A Discriminative Approach for Unsupervised Clustering of DNA Sequence Motifs

**DOI:** 10.1371/journal.pcbi.1002958

**Published:** 2013-03-21

**Authors:** Philip Stegmaier, Alexander Kel, Edgar Wingender, Jürgen Borlak

**Affiliations:** 1 Biobase GmbH, Wolfenbüttel, Germany; 2 geneXplain GmbH, Wolfenbüttel, Germany; 3 Universätsmedizin Göttingen, Abteilung Bioinformatik, Göttingen, Germany; 4 Medizinische Hochschule Hannover, Zentrum für Pharmakologie und Toxikologie, Hannover, Germany; Ottawa University, Canada

## Abstract

Algorithmic comparison of DNA sequence motifs is a problem in bioinformatics that has received increased attention during the last years. Its main applications concern characterization of potentially novel motifs and clustering of a motif collection in order to remove redundancy. Despite growing interest in motif clustering, the question which motif clusters to aim at has so far not been systematically addressed. Here we analyzed motif similarities in a comprehensive set of vertebrate transcription factor classes. For this we developed enhanced similarity scores by inclusion of the information coverage (IC) criterion, which evaluates the fraction of information an alignment covers in aligned motifs. A network-based method enabled us to identify motif clusters with high correspondence to DNA-binding domain phylogenies and prior experimental findings. Based on this analysis we derived a set of motif families representing distinct binding specificities. These motif families were used to train a classifier which was further integrated into a novel algorithm for unsupervised motif clustering. Application of the new algorithm demonstrated its superiority to previously published methods and its ability to reproduce entrained motif families. As a result, our work proposes a probabilistic approach to decide whether two motifs represent common or distinct binding specificities.

## Author Summary

Transcription factors play a central role in the regulation of gene expression. Their interaction with specific elements in the DNA mediates dynamic changes in transcriptional activity. Databases store a growing number of known DNA sequence patterns, also denoted as DNA sequence motifs that are recognized by transcription factors. Such databases can be searched to find a match for a newly discovered pattern and that way identify the potential binding factor. It is also of interest to cluster motifs in order to examine which transcription factors have similar binding properties and, thus, may promiscuously bind to each other's sites, or how many distinct specificities have been described. To gain deeper insight into the similarities between DNA sequence motifs, we analyzed a comprehensive set of known motifs. For this purpose we devised a network-based approach that enabled us to identify clusters of related motifs that largely coincided with grouping of related TFs on the basis of protein similarity. On the basis of these results, we were able to predict whether two motifs belong to the same subgroup and constructed a novel, fully-automated method for motif clustering, which enables users to assess the similarity of a newly found motif with all known motifs in the collection.

## Introduction

An important goal of biological research is to understand the mechanisms that control gene expression. Of key interest are transcription factors (TFs) that bind to specific functional elements in the DNA and from there regulate expression of target genes. Binding site sequences recognized by individual TFs often exhibit distinct patterns of more or less stringent nucleotide preferences at different positions, also denoted as DNA sequence motifs. There are commercial and public databases like Transfac® (public or commercial) [1] and Jaspar (public) [2] that maintain libraries of DNA sequence motifs in the form of Position-specific Frequency Matrices (PFMs). The PFM is a 4×L matrix whose columns describe nucleotide preferences at corresponding binding site positions by their absolute or relative frequencies.

In recent years there has been increased interest in methods to quantitatively compare DNA sequence motifs. There are two eminent applications for such methods in the current literature. One is to search a library of known motifs with a newly discovered pattern to check its novelty or to derive hypotheses about TF families that could be assigned to the search pattern. This database search application is of increasing importance for the widely adopted ChIP-seq and ChIP-chip assays that enable computational extraction of DNA sequence motifs from large sets of genomic regions bound by a transcription factor of interest [3], [4]. In the second application, quantitative comparison forms the basis to define groups or families of motifs. The growing body of known binding motifs for different transcription factors has stimulated interest to assign patterns to groups representing distinct specificities. While DNA sequence motifs in databases are typically defined for a narrow selection of proteins such as a group of isoforms, a subfamily or a complex, motif families may widen the scope to represent the DNA-binding properties, e.g., of a whole class of transcription factors.

A number of methods have been developed for motif comparison. Kielbasa et al. [5] proposed a combination of Chi^2^ distance and correlation coefficients of Position-specific Weight Matrix (PWM) scores to group highly similar binding specificities. Mahony et al. [6] compared global and local alignment algorithms as well as column-wise similarity metrics with respect to their ability to recognize motifs belonging to the same transcription factor class and developed methods to cluster PFMs into representative Familial Binding Profiles (FBPs) [7]. By now, many tools are available for motif comparison and clustering such as MatCompare [8], STAMP [6], [9], T-Reg Comparator [10], MATLIGN [11], Tomtom [12], Mosta [13], or KFV [14].

A large group of methods compares motifs on the basis of column-wise scores that scale the similarity or dissimilarity of aligned motif positions. Column-wise scores that have been described for DNA sequence motif analysis include Chi^2^ statistics [5], Kullback-Leibler divergence [10], Pearson correlation [6], Fisher-Irwin test P-values [8], absolute, squared or Euclidean distances [15], [7], [12], generalized log-odds scores [16], [17], Bayesian methods [18], or fuzzy integral techniques [19]. One advantage of column-wise scoring is its straightforward application within standard local or global alignment algorithms, e.g. [6]. Other methods assess motif similarity on the basis of how binding sites are predicted by corresponding PWMs. Similar to the score correlation approach described in [5], the Mosta algorithm analyzes the tendency of binding sites to overlap when they are predicted with two PWMs at a certain score threshold and for a certain background distribution of nucleotides [13]. Finally, the alignment-free KFV method evaluates the similarity of fixed-length k-mer vectors to which motifs are converted [14]. In this work we present the information coverage (IC) criterion as a further enhancement of column-wise scoring. The IC evaluates the fraction of information of compared motifs that is covered by an alignment. Alignments between related and unrelated motifs exhibit different IC distributions. Combination of the IC with existing motif alignment scores improved their motif classification performance.

Despite the great interest in classification and clustering of DNA sequence motifs, little progress has been made to define families of motifs that methods aim to identify. Validation of motif clustering results mainly addressed their homogeneity with respect to structural classes of TFs, such as ETS, homeobox or nuclear receptor proteins. On the other hand, inference of clusters relied on ad-hoc cut-offs to prevent potential false merges of PFMs into common groups or cut hierarchical clustering trees at an optimal height that balanced inter- and intracluster variability, see e.g. [6], [13], [14]. Neither of these strategies used information about known motif families to define such thresholds.

In this work we therefore undertook a first step to compile a comprehensive collection of motif families that can be used as a goal set for motif clustering methods. We denote as motif family a (sub)set of motifs from the same TF class with a common, distinct binding specificity. Methods developed in this work aimed at identifying clusters of motifs that correspond to such motif families and to propose a representative FBP. Our analyses used a set of 1001 Transfac matrices that were assigned to 35 motif classes mainly corresponding to distinct classes of DNA-binding protein domains [20]–[22]. To subgroup them into motif families, we next devised a network analysis-approach. This procedure constructed networks of Transfac matrices that revealed families of similar motifs as modules of highly connected nodes. Computational graph-cluster analysis confirmed our manual observations based on network visualizations. Furthermore, we examined the concordance between extracted motif clusters and phylogenies of corresponding DNA-binding domains as well as experimental knowledge regarding specificities of certain types of transcription factors. According to this assessment, the motif clusters matched protein domain families as well as prior expectations about DNA-binding properties of some well-described transcription factors. A set of motif families assembled on the basis of network analysis results was then applied to train a probabilistic classifier. The classifier was designed to assign a probability to the hypothesis that two PFMs belong to the same motif family given their similarity score and offers a natural decision threshold. We integrated the new classification function into a novel algorithm for unsupervised motif clustering and demonstrate its ability to extract meaningful motif clusters that are represented by Familial Binding Profiles.

Our workflow for the general goal of clustering DNA sequence motifs depicted in this article can be summarized as follows. We first describe novel information coverage-scores and their validation. We then illustrate the use of the best score for further analysis of motif networks and extraction of motif families. Finally, we report on the development and validation of a new probabilistic classifier that enabled us to conduct motif clustering in an unsupervised fashion and accurately reproduced the entrained motif families.

## Results

### Improvement of motif similarity scores by augmentation with information coverage

**Figure 1:**
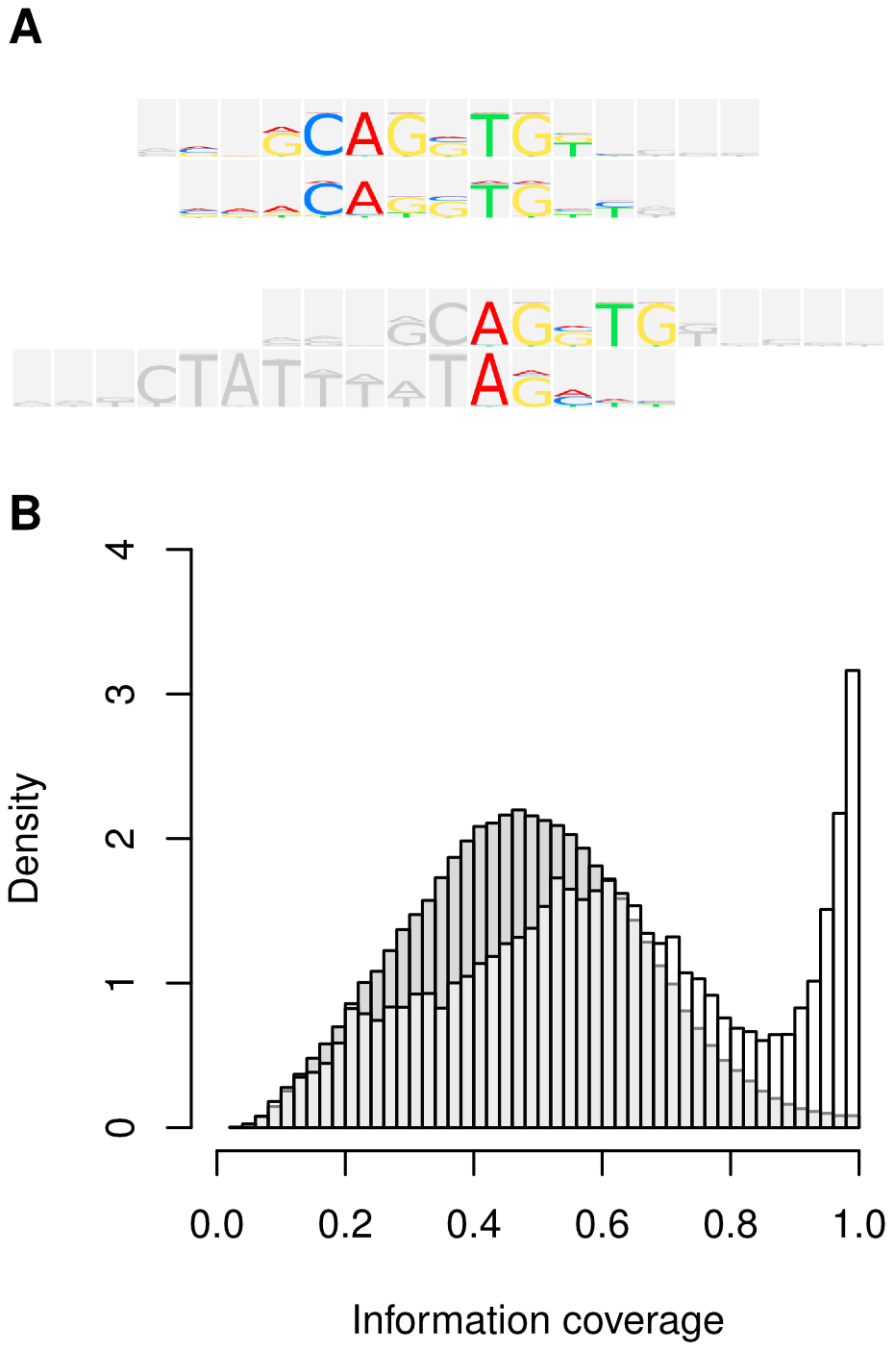
Intra-class alignments cover a higher fraction of motif information than inter-class alignments. (A) Example alignments illustrate the information coverage (IC) criterion. Depicted are m2match outputs of an intra-class alignment for two TFs of the BHLH class E47 and MyoD (top) and an inter-class alignment for the E47 motif and the PFM of MADS transcription factor RSRF (bottom). (B) Histograms of IC values observed in intra-class and inter-class alignments. Alignments were selected using the Euclidean distance (ED) score and information coverage was calculated using the *sqr* formula (Material and Methods). In total there were 436080 inter-class and 64420 intra-class alignments. Intra-class alignments showed a tendency for higher IC than inter-class alignments and specifically exhibited a pronounced peak at high IC values which is absent in the inter-class distribution.

Our motif alignment program *m2match* [17] was designed to search for pairwise ungapped local alignments between PFMs. The algorithm selects an optimal alignment according to the score which is the sum of individual column-column scores (column-wise scoring). For this study we developed new composite scores that integrate an alignment feature denoted as *information coverage*. Information coverage refers to the fraction of information of the motifs that is covered by their alignment. The information of a DNA sequence motif is determined by probability distributions over nucleotides in each of its positions. Figure 1A shows alignments with different information coverage. The alignment of basic helix-loop-helix (BHLH) matrices for transcription factors E47 and MyoD (Fig. 1A top) reaches out over most of the informative positions, whereas the (local) alignment of the E47 motif with a PFM for the MADS transcription factor RSRF (Fig. 1A bottom) omitted several informative positions (gray logo positions). In our study set from the Transfac database alignments between matrices of the same class (intra-class alignments) exhibited a pronounced peak at high IC values which is absent in the IC distribution obtained from inter-class alignments (Fig. 1B).

We subsequently derived new scores that take into account the information coverage of alignments. The new scores extend previously described Euclidean distance (ED) [12] and sum-of-squared distance (SSD) [7] metrics by information coverage terms and are straightforward to compute. Specific variants implemented in m2match are denoted as ED.ave, ED.sqr, SSD.ave, and SSD.sqr (Material and Methods). We carried out a comparison of existing and new methods with respect to two different performance statistics as well as two different libraries of PFMs, Transfac and Jaspar [1], [2].

**Figure 2:**
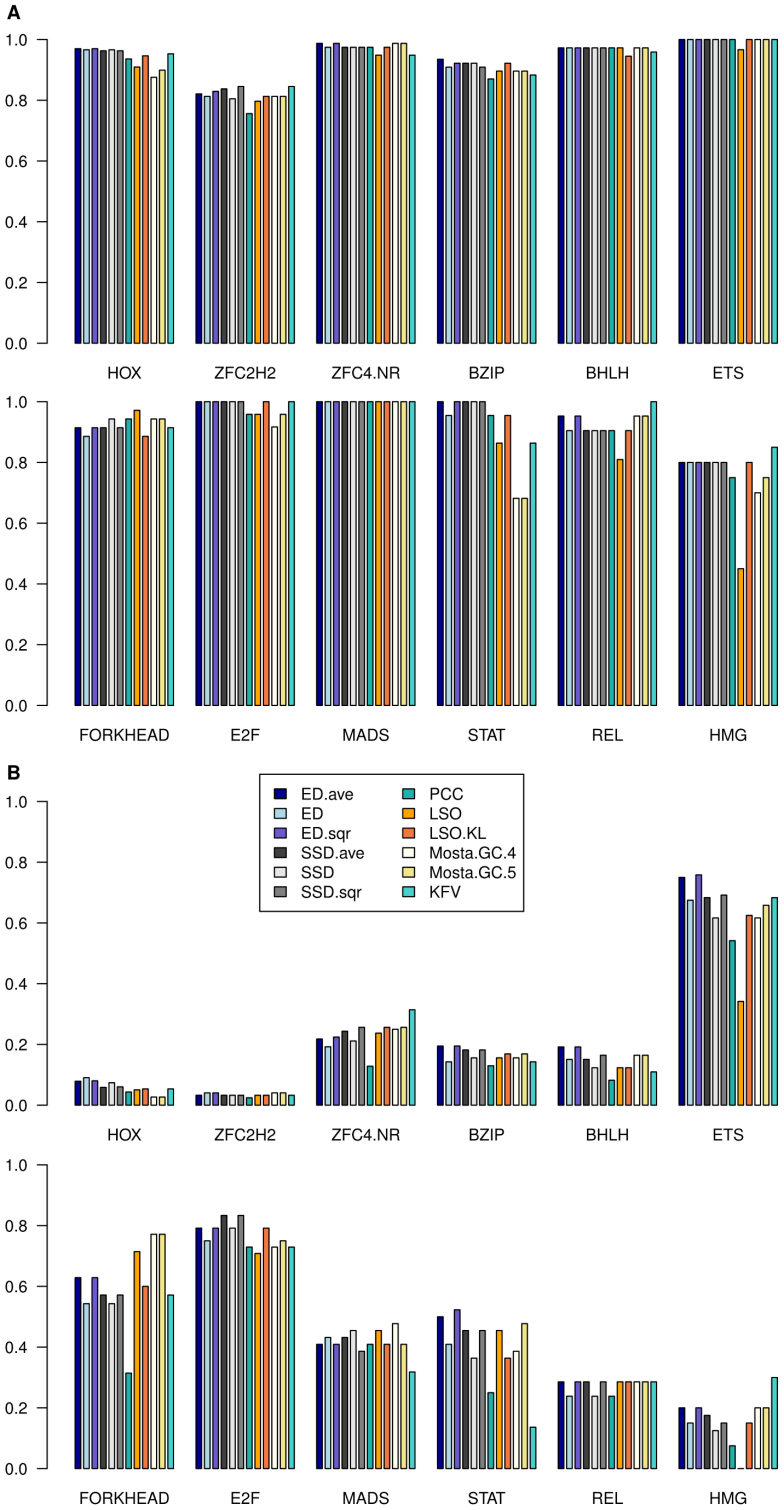
Best hit and class-depth statistics achieved by different methods. The plots cover the 12 largest classes of the Transfac set with at least 20 motifs. Each bar group represents one motif class. (A) Best hit (B) Class-depth.

Figure 2 shows best hit and class-depth statistics achieved by different methods for the 12 largest Transfac classes with at least 20 PFMs. Overall, integration of IC indeed improved ED as well as SSD scores, with *ave* and *sqr* variants showing similar performance. Differences were rather small according to the best hit assessment. The ability to recognize other class members increased most strongly with regard to the class-depth statistic where differences up to 5% were recorded for the median values (see also Table 1 below). In few cases, e.g. in the homeobox (HOX) or MADS classes, the ED score was slightly better than ED.ave and ED.sqr scores according to class-depth. However, the improvements visibly outweigh minor performance decreases. Best hit statistics for SSD.ave and SSD.sqr scores were similar or slightly worse than for the SSD score, whereas consideration of IC again improved class-depth statistics in most classes. Some score methods excelled on some classes, but at the same time exhibited difficulties with other classes. For instance, Mosta did not perform as well as other methods on the STAT class according to best hits, and on the HOX class according to class-depth, but the method was ahead on the FORKHEAD class according to class-depth. In contrast, we observe that results of the IC-extended ED and SSD scores were consistently at a high level without bearing remarkable weaknesses for particular TF classes.

**Table 1:** Best hit and class-depth statistics obtained with different methods on the set of classified Transfac PFMs.

	Best hit			Class depth (Med)			Class depth (Lqr)			Class depth (Uqr)			
Method	Top 5	Min 20	Min 10	Top 5	Min 20	Min 10	Top 5	Min 20	Min 10	Top 5	Min 20	Min 10	Average
ED.ave	**0.94**	**0.95**	**0.95**	0.14	**0.36**	**0.39**	**0.07**	**0.26**	**0.30**	0.27	**0.46**	**0.49**	**0.62**
ED	0.93	0.93	0.93	0.12	0.32	0.34	0.05	0.21	0.22	0.24	0.42	0.44	0.59
ED.sqr	**0.94**	**0.95**	**0.95**	**0.15**	**0.36**	**0.39**	**0.07**	**0.26**	**0.30**	0.27	**0.46**	**0.49**	**0.62**
SSD.ave	0.93	0.94	0.94	0.13	0.34	0.37	**0.07**	0.23	0.29	0.27	0.45	**0.49**	0.61
SSD	0.93	0.94	0.94	0.12	0.31	0.34	0.05	0.19	0.20	0.24	0.41	0.43	0.60
SSD.sqr	0.93	0.94	0.94	0.14	0.34	0.38	**0.07**	0.23	0.29	0.27	0.45	**0.49**	0.61
PCC	0.90	0.92	0.90	0.08	0.25	0.24	0.04	0.14	0.13	0.17	0.36	0.35	0.55
LSO	0.90	0.88	0.84	0.12	0.30	0.32	0.04	0.17	0.20	0.24	0.39	0.40	0.56
LSO.KL	0.92	0.93	0.92	0.13	0.32	0.33	0.06	0.22	0.24	0.24	0.42	0.43	0.59
Mosta.GC.4	0.91	0.89	0.90	0.13	0.34	0.37	**0.07**	0.21	0.24	0.22	0.44	0.46	0.59
Mosta.GC.5	0.91	0.90	0.91	0.13	0.35	0.38	**0.07**	0.22	0.25	0.24	**0.46**	0.47	0.60
KFV	0.92	0.93	0.94	0.13	0.31	0.34	0.06	0.17	0.21	**0.30**	0.42	0.45	0.59

Values were summarized for different subsets of motif classes. Top 5: the five largest classes; Min 20: classes with at least 20 members; Min 10: classes with at least 10 members. For the class depth averages of upper (Uqr) and lower quartiles (Lqr) as well as medians (Med) are given. Highest values in each column are highlighted in bold. The *Average* summarizes the six leftmost columns.

Table 1 summarizes our results on the Transfac data set for different sets of motif classes. The values show that inclusion of the information coverage led to an overall improvement of ED scores, especially according to class-depth. Based on the summary values, results were similar for SSD scores, but inclusion of IC did not accomplish as strong improvements as for ED scores. Average values over the six leftmost columns confirm that ED.ave and ED.sqr scores achieved the best overall performance among all of the compared methods.

The strongest methods of the previous comparison were selected to further compete on the Jaspar CORE database. Here we calculated best hit and class-depth statistics for the five largest Jaspar families as well as the Jaspar families with at least 10 motifs, including the zinc finger family (see Material and Methods). Results are summarized in Table 2. As for the Transfac data set, integration of information coverage improved motif classification by ED and SSD scores and the extended scores were competitive to the other state-of-the-art methods. Notably, the advantage of SSD.ave and SSD.sqr scores over the SSD score is more pronounced on the Jaspar data set than on the Transfac collection. On the set of families with at least 10 motifs, the ED.sqr achieved a 6% better performance than the ED score with respect to class-depth. Again ED.sqr and ED.ave scores attained highest average values over best hit and class-depth criteria (Table 2), which is in concordance with the Transfac results. We therefore carried out further analysis of motif relationships using m2match with the ED.sqr score.

**Table 2:** Best hit and class-depth statistics obtained with different methods on the Jaspar CORE set.

	Best hit		Class-depth (Med)		Class-depth (Lqr)		Class-depth (Uqr)		
Method	Top 5	Min 10	Top 5	Min 10	Top 5	Min 10	Top 5	Min 10	Average
ED.ave	**0.80**	0.77	0.18	0.23	0.02	0.09	0.30	**0.34**	**0.50**
ED	0.79	0.78	0.14	0.18	0.02	0.08	0.27	0.32	0.47
ED.sqr	**0.80**	**0.78**	0.18	**0.24**	0.02	**0.10**	**0.31**	**0.34**	**0.50**
SSD.ave	**0.80**	**0.78**	0.15	0.21	0.02	0.09	0.27	0.32	0.48
SSD	0.78	0.76	0.10	0.16	0.02	0.07	0.23	0.30	0.45
SSD.sqr	**0.80**	**0.78**	0.15	0.21	0.02	0.09	0.27	0.32	0.49
Mosta.GC.4	0.77	0.72	0.16	0.22	0.02	0.09	0.27	**0.34**	0.47
Mosta.GC.5	**0.80**	**0.72**	**0.20**	**0.24**	**0.03**	**0.10**	0.27	0.33	0.49
KFV	0.77	0.77	0.16	0.21	0.01	0.09	0.26	0.33	0.48

Values were summarized for different subsets of motif classes. Top 5: the five largest classes; Min 10: classes with at least 10 members. For the class-depth averages of upper (Uqr) and lower quartiles (Lqr) as well as medians (Med) are given. Highest values in each column are highlighted in bold. The *Average* summarizes the four leftmost columns.

### Motif network analysis

Network analysis was applied to further split motif classes into clusters of closely related binding specificities. We compiled networks connecting each motif with other class members that achieved a higher score than non-class members. Finally, we applied the Markov Clustering Algorithm (MCL) [23] to each motif network containing at least 5 motifs. This network-based approach was motivated by our class-depth analysis. The class-depth statistic assumed distinct, motif class-specific levels across methods that participated in the comparison (Fig. 2). For instance, class-depth values were below 20% in the two largest classes, HOX and C2H2 zinc fingers (ZFC2H2), whereas most methods achieved a class-depth over 50% for the classes ETS, FORKHEAD, and E2F. However, the four smallest classes STAT, MADS, REL, and HMG were associated with lower values (Fig. 2B), which rules out that class-depth levels depended on motif class sizes. We conjectured that these class-specific levels originated from the existence of motif families that formed subgroups of highly similar matrices within classes.

Network analysis predicted in total 125 and 135 clusters (including disconnected singletons) when using ED.sqr or ED scores, respectively (Table S1). No connections between matrices were obtained in the ATHOOK class (Table S2). In ten TF classes comprising 6 to 60 matrices all PFMs were drawn together into a single cluster, not taking into account disconnected motifs (Table S2). These classes encompass well-characterized TF classes such as basic helix-span-helix (BHSH), ETS, FORKHEAD, or GATA zinc fingers (ZFGATA). Networks of another ten classes were each split into two clusters by MCL (Table S3). Finally, between 2 and 12 clusters were identified in the classes REL, basic leucine-zipper (BZIP), BHLH, nuclear receptor zinc fingers (ZFC4-NR), ZFC2H2, and HOX. Thus, C2H2 zinc finger and homeobox classes exhibited an outstanding number of different binding specificities, whereas other TF classes comprised much fewer different motif types (1–3 without singletons, 1–6 with singletons).

We compared motif network clusters to phylogenies of DNA-binding domains for the classes BHLH, BZIP, HMG, MADS, REL, SMAD, STAT and ZFC4-NR. A detailed discussion of several of these classes is provided in the supplement (Text S1). Overall, the extracted motif clusters were closely correlated with subtypes of DNA-binding domains. Strongest departures between motif clusters and protein domain phylogenies were observed in BZIP and STAT classes and, according to our assessment, induced by different types of spacers or different numbers of half-sites covered by PFMs (Text S1).

**Figure 3:**
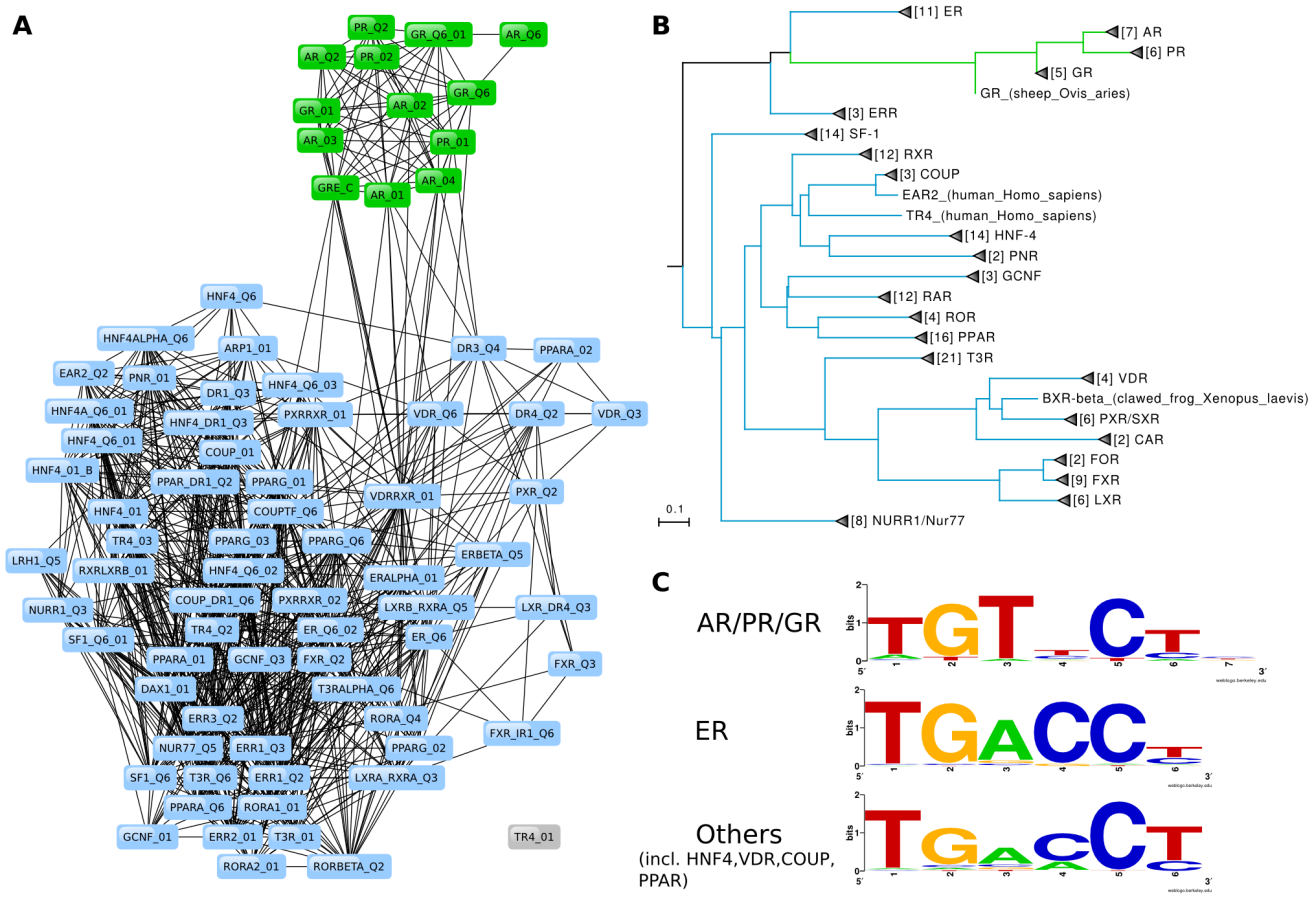
Motif network and DNA-binding domain phylogeny for the ZFC4-NR class. (A) Motif network of nuclear receptor motifs with colors indicating clusters extracted by MCL. (B) Phylogeny of nuclear receptor DNA-binding domains represented by matrices in the motif network. Branch colors correspond to MCL clusters in A. (C) Motif logos were generated using WebLogo [33] for binding sites of NR3C proteins (top), estrogen receptor (middle), and nuclear receptors from other families (bottom). The half-site logos illustrate that estrogen receptor motifs were correctly clustered separately from NR3C matrices and with the other nuclear receptors.

Motif clusters often correlated with broader protein families or subfamilies such as BHLH-Zip, CREB/ATF, SMAD factors in BHLH, BZIP and SMAD classes, respectively. SREBP matrices in the BHLH class and 3-Ketosteroid receptors of the nuclear receptor class presented exceptions to this trend. In compliance with the dual binding specificities of SREBP [24], network analysis assigned its motifs to two clusters, with one reserved exclusively for the SREBP-specific pattern. In the nuclear receptor class, motif clusters accurately distinguished the half-site specificity of 3-Ketosteroid (NR3C) receptors from other nuclear receptors, whereas the protein phylogeny reflects the standard grouping of Estrogen and Estrogen-related receptors (NR3A and NR3B) with those of the NR3C type [25] (Fig. 3). However, half-sites recognized by NR3A and NR3B proteins resemble the pattern bound by non-NR3 receptors and therefore Estrogen receptor matrices were allocated in one cluster with PFMs of non-NR3 receptors (Fig. 3). The molecular causes of different DNA-binding preferences within the nuclear receptor class have been described in detail by Zilliacus et al. [26].

In summary, the network-based analysis delivered meaningful results for a wide range of transcription factor classes. Also in the large and diverse HOX and ZFC2H2 classes the method proposed groups of motifs dominated by closely related transcription factors. In addition, some cases could be highlighted where computational predictions accurately fit prior experimental knowledge such as for SREBP factors or nuclear receptors.

### A discriminative classifier for motif families

In the following we used clusters of Transfac matrices derived through motif network analysis to train a classifier for motif families. It was the ultimate goal of our study to predict common motif family membership purely by computational means. The conceived classifier accomplished this on the basis of the motif similarity score without requiring information about TF classes. For this we compiled a list of 47 Transfac matrix sets for 26 motif classes (Table S4). These were used as representatives of motif families for the classifier training. Some minor modifications were made to the raw MCL clusters in order to omit some potential false positive or uncertain cluster members which are described in the supplement. For instance, we discarded the V$NMYC_02 matrix that was falsely assigned to the BHLH-only cluster.

**Figure 4:**
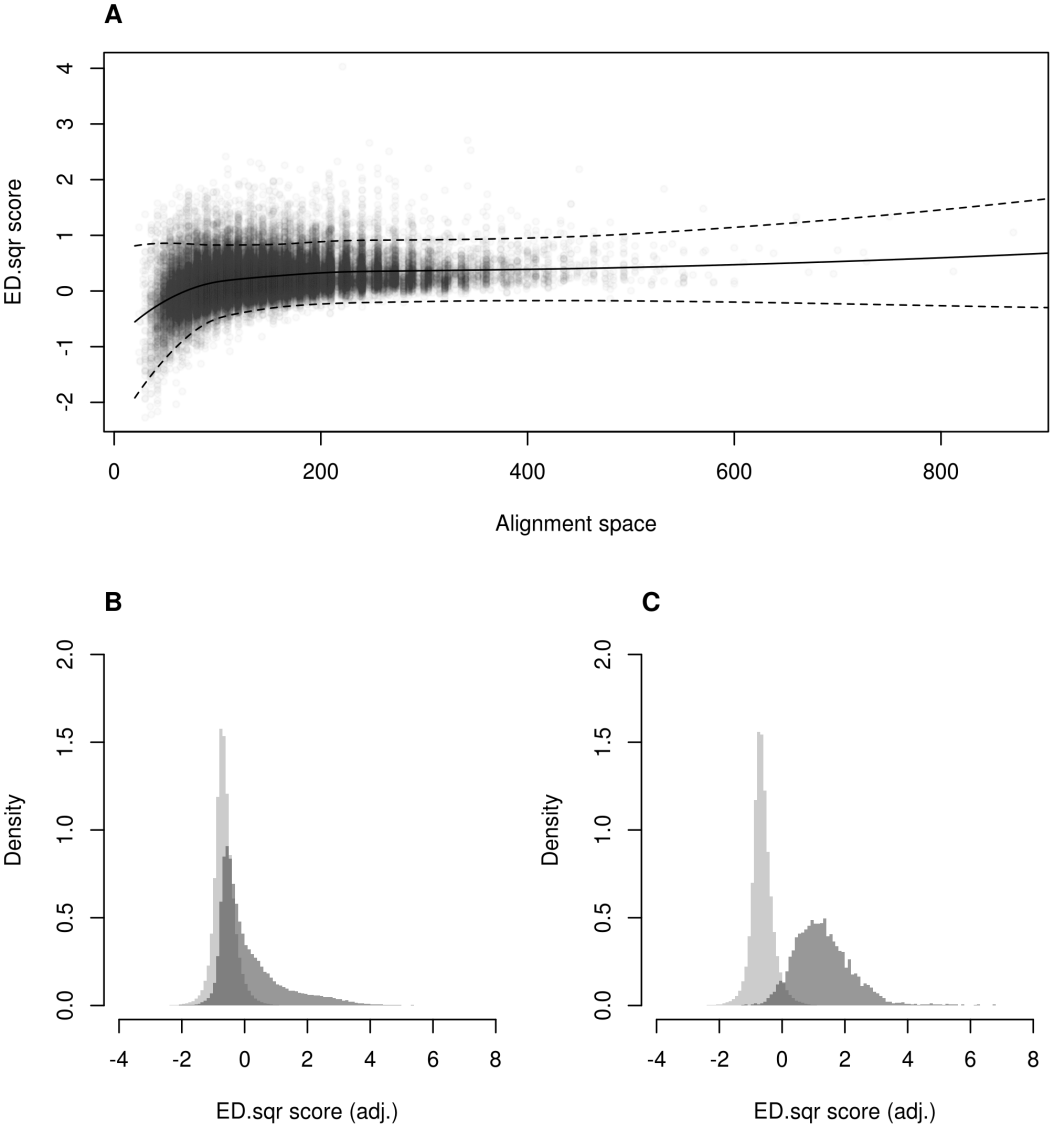
ED.sqr scores for inter-class, intra-class, and intra-family alignments. (A) Scatter plot of ED.sqr scores and alignment space values observed in inter-class alignments. The alignment space was the product of aligned motif lengths, which is proportional to the number of possible alignments. Curves show conditional mean and variance estimates (2σ above and below the mean) obtained with non-parametric regression. (B) Histograms of adjusted ED.sqr scores for inter-class (light) and intra-class alignments (dark). (C) Histograms of adjusted ED.sqr scores for inter-class (light) and intra-family alignments (dark).

To make alignment scores for PFM pairs of different lengths comparable we estimated the dependence of mean and variance of inter-class scores on the space of possible alignments (Fig. 4A). Raw ED.sqr scores were subsequently adjusted according to the following formula:
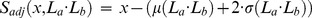
(1)In (1), x is the raw score, L_a_ and L_b_ are the lengths of matrices a and b, μ() is the conditional mean and σ() is the conditional standard error estimated by non-parametric regression. Next we compared distributions of intra-class scores, intra-family and inter-class scores (Fig. 4B+C). While distributions of intra- and inter-class scores strongly overlapped (Fig. 4B), the intra-family distribution exhibited a smaller overlap with and a different mode than the inter-class distribution (Fig. 4C). To utilize this information in a classification framework, we trained a logistic regression model with positive examples comprising intra-family alignment scores and with inter-class scores as negative examples. We considered inter-class instead of inter-family scores as a careful choice for the negative set. This follows from our results of motif network analysis where we also used inter-class scores to place or omit edges between PFMs. Hence, for inter-class alignment scores we were certain that they belonged to pairs of unrelated motifs. The resulting classifier estimates the probability that two matrices belong to the same motif family (F = 1) given the adjusted score of their alignment:
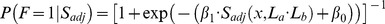
(2)The logistic regression (LR) classifier is both discriminative and probabilistic. The approach moreover provides for a natural threshold of P(F = 1|S_adj_)>50% to decide in favor of the hypothesis that compared motifs belong to the same family. Parameter estimates reported by R's glm function [27] for adjusted ED.sqr scores were β_1_ = 5.294,β_0_ = −3.3296.

### A novel method for unsupervised motif clustering

We incorporated the classification function into a novel motif clustering algorithm as part of m2match. The goal of motif clustering is to identify a non-redundant set of Familial Binding Profiles by clustering a given collection of motifs [6], [7]. In the context of our work we can now formulate the more precise objective that the motif clusters inferred by the algorithm shall match defined motif families. Hence, the motif clusters and respective FBPs are predictions about motif family assignments. Our algorithm accomplished this as follows. For a set of TF matrices m2match first calculated a distance matrix for subsequent agglomerative clustering. Off-diagonal entries of the distance matrix were set to complementary motif family probabilities (1-P(F = 1|S_adj_)). The distance matrix was applied in hierarchical average-linkage clustering. During the clustering process each cluster was represented by a Familial Binding Profile, where the input set of TF matrices was regarded as initial set of FBPs. At each clustering step, the program examined whether the alignment score of the FBPs representing two clusters satisfied the motif family threshold. Furthermore it was tested that a newly formed FBP detected all original TF matrices above the same cut-off. The motif family threshold was set to the natural classification threshold P(F = 1|S_adj_)>50%. A merging was considered valid if it satisfied the described criteria and the new FBP subsequently replaced the two FBPs from which it had been derived. If either of the two criteria was not satisfied a cluster and its representative FBP were marked as invalid and could not further contribute to forming valid clusters. A new FBP was derived from the alignment of two predecessor FBPs based on a weighted average of aligned matrix positions. Empty (unaligned), flanking positions of the alignment were filled with uniform nucleotide distributions and were assigned a weight of 1. The weight of real matrix positions was the square root of the number of underlying binding site sequences. We imposed a maximum of 200 for the number of binding site sequences to be taken into account in order to accommodate ChIP-assay derived matrices, whose underlying binding site alignment may sometimes cover several hundred or over a thousand genomic sites. This maximum was therefore adopted to prevent matrices derived from a very large number of binding sites from overriding the contribution of other matrices to the FBP.

**Figure 5:**
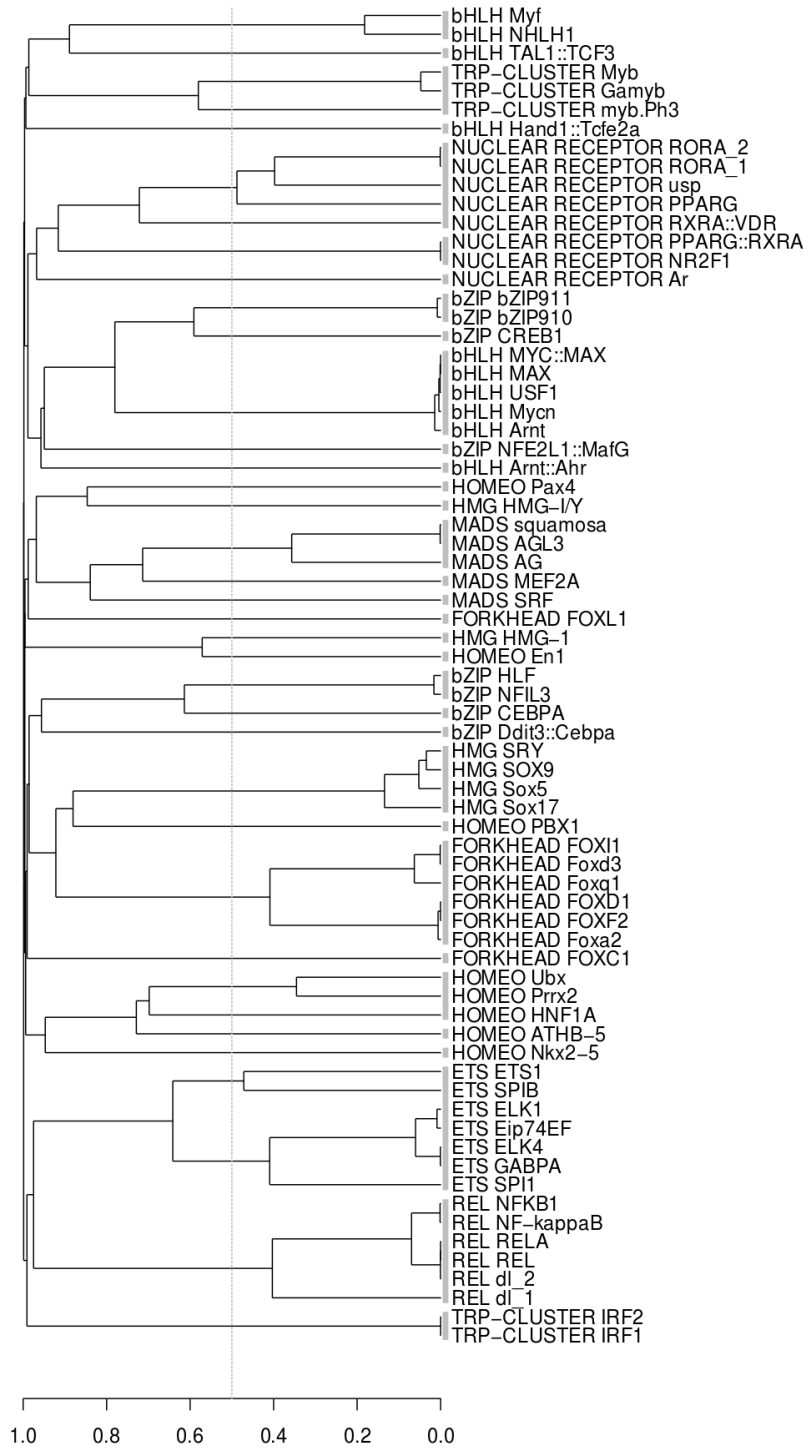
Clustering of 71 non-zinc finger motifs from Jaspar. Gray boxes between dendrogram and matrix names indicate motif clusters. The dotted line points out the 50% motif family threshold. Some clusters were merged below that threshold, because FBPs formed in the course of the clustering process provided for a better presentation of the motif family than the basic motifs.

Figure 5 shows the clustering result for the set of 71 non-zinc finger Jaspar motifs that was also used in previous studies, see e.g. [14]. The set was split into 33 FBPs by m2match using the ED.sqr score. This is higher than reported in other studies, where the number of 16 FBPs was obtained in [14]. A striking difference between our method and those of previous studies is that all motif families returned by m2match were homogeneous with respect to the TF class. One reason why other methods achieved fewer clusters is therefore that merges happened between motifs from different TF classes. To our knowledge no other method has before produced a clustering of this particular data set with perfect class homogeneity. Moreover, the FBPs formed from multiple Jaspar PFMs and several of the matrices that m2match left as singletons correspond to separate motif families as identified in the course of our motif network analysis. For instance, MCL extracted separate clusters comprising PBX-, NKX-, and PAX-type motifs, which we observe as singletons in the Jaspar clustering result. In both the Transfac and Jaspar PFM set, MEF2 and SRF motifs formed separate motif families (Table S3). Interestingly, plant MADS matrices of the Jaspar set were assigned to one further FBP (Fig. 5). The Transfac PFMs collection used in this study consisted exclusively of matrices for vertebrate transcription factors and did not contain any plant motifs. For comparison, Mosta merged all Jaspar MADS matrices into a single cluster [13]. The FBP of SOX/SRY-type motifs (Fig. 5) matches the results of our analysis of Transfac HMG motifs. We think that it is plausible to assign the Jaspar HMG-1 matrix to a separate motif family as suggested by m2match, because, unlike HMG box factors of the SOX/SRY-type, the sequence specificity of the HMG-1 factor was shown to be limited to oligomers enriched in certain dinucleotides [28]. The androgen receptor matrix Ar was not clustered with other nuclear receptor motifs, since it was the only member of the NR3C family in this data set. Finally, m2match faithfully grouped all the ETS and REL motifs of the Jaspar set into one FBP for each class, which again correlates perfectly with prior results on the Transfac data set and was not achieved by some other previously published methods (clustering of REL dl_1 reported in [14]). Note that Jaspar REL motifs encompassed only the Rel/NF-kappaB subset, so that allocation of their PFMs into one single FBP agrees with our earlier results.

We also applied our method to the motif classes of the Transfac study set. A summary of the results is provided in Table S1. For comparison, we also included results obtained with ED scores. Rand indexes for the clusterings by network analysis and by m2match show that m2match was able to closely reproduce the network cluster results, achieving a Rand index of about 91% on average (Table S5). Among the classes with lowest agreement between the methods, m2match joined IRF or RUNT matrices into one single FBP for each class, whereas the TBP class was split into two clusters with the ATATA_B matrix as singleton (see also Table S3). Conversely, m2match partitioned the group of non-NR3C motifs within the nuclear receptor class into several smaller clusters, but otherwise assigned all NR3C motifs to a common group with the exception of V$AR_Q6 (Figure S4). Generally, we observe that the hierarchical clustering approach had a tendency to produce more motif clusters than MCL applied to motif networks, especially in the large HOX and ZFC2H2 classes. In the HOX class, m2match perfectly recovered the IRX motif family. Other training motif families were partially restored (Table S6). Furthermore, the method detected FBPs predominated by matrices for certain protein subfamilies which selected for HNF1-, PBX-, or SIX-type motifs, respectively. In the ZFC2H2 class, m2match re-identified all eight motif families. The program assigned one more Helios A matrix (V$HELIOSA_02) to family #40 (Table S4) and predicted new clusters with high protein subfamily-homogeneity that comprised EGR motifs or ZIC and GLI matrices (Table S7), which were part of one large cluster in the MCL/motif network result (Text S3). The ETS and FORKHEAD classes show that the algorithm is able to detect that a motif set consists largely of one single FBP, albeit it did not to join the FOXO1 matrix with the large FORKHEAD cluster (Figure S4). FBPs inferred for BHLH and BZIP classes also closely resembled motif clusters identified during network-based analysis. Matrices for AHR factors were not allocated with other BHLH-Zip motifs but formed a separate FBP, separating the CACGCG-consensus of AHR motifs from the CACGTG-consensus of other E-boxes in the BHLH-Zip group. In addition, the selectivity for a particular factor subfamily suggests that this finding is biologically meaningful. In the BZIP class our method produced new clusters of Maf-type matrices and of VBP/HLF/E4BP4 matrices. Several matrices previously assigned to larger groups were isolated. These comprise unclear or false assignments in clusters derived from motif networks, e.g. V$CEBP_01, V$DBP_Q6, and V$TAXCREB_02, so that we regard their separation from other motifs as an improvement of the previous solution.

## Discussion

This study developed novel solutions for some important problems in motif classification and clustering. First, we presented novel motif similarity scores that make use of the information coverage criterion and showed improved performance in retrieving related motifs of the same class. Then, two new methods for clustering of DNA-sequence motifs were developed, one network-based approach and one based on hierarchical clustering. Both motif clustering methods demonstrated their ability to propose motif clusters that were biologically meaningful as validated with respect to protein domain phylogenies and prior knowledge about distinct binding specificities.

An important aspect of the IC extension is its evaluation of a local alignment as a whole. In the presented formulations it is not restricted to distance metrics used in this work, but can be combined with other alignment scores as well. This development therefore motivates exploration of further possibilities to improve motif alignment scoring apart from improving column-wise scoring metrics.

It was previously noted that some scoring methods can report high scores for aligned PFM positions regardless of their information content [18], [19]. This induces a potential source of false positives, because it is disregarded whether aligned positions confer specificity. Column-wise scores based on Bayesian and fuzzy integral approaches have been developed that did not suffer from that flaw [18], [19]. Also the LSO score has the property of assigning less extreme scores to less informative positions [16]. On the contrary, ED and SSD metrics do not differentiate between PFM columns with respect to their information content. Although the IC criterion was conceived from the perspective of distinguishing between intra- and inter-class alignments, it also addresses the handling of informative and non-informative columns. In contrast to other solutions our treatment of information coverage did not directly reduce the contribution of less or non-informative motif positions to an alignment score, but was designed to favor alignments extending over as much information of compared motifs as possible. It is therefore in our interest to further explore IC as an alternative or additional strategy to attribute more importance to informative motif positions.

Motif network analysis enabled us to compile a set of motif families, which were required as input for subsequent classifier training. This part of our study highlighted the diversity among C2H2 zinc finger and homeobox motifs. We think that further study of the causes of the exceptional positioning of these classes as well as the relative homogeneity with regard to the number of different binding specifities in other classes can elucidate new aspects of the evolution of cellular regulatory systems. Furthermore, inspection of motif clusters and corresponding protein phylogenies showed that distinct binding patterns can appear at different levels of primary sequence divergence. It is of great interest to identify the changes necessary to generate a new binding specificity within a transcription factor class and the results of our study can be explored in that direction. As a computational tool, the network-based analysis of motif clusters was not purely unsupervised, because it used information about class membership. In practice, this is not a significant burden as the classes of PFMs collected in large databases are usually known. As a particular advantage, the devised method did not require any further choice of parameters (MCL was invoked with default parameters).

The motif families derived by network analysis enabled us to develop another novel approach for motif clustering on the basis of the logistic regression model. Important novelties of this method are its discriminative training with positive and negative examples of motif alignment scores and its integration of a probabilistic decision threshold. Specifically, at the natural 50% threshold the devised algorithm was able to produce meaningful motif clusters. Therefore, unlike other methods it can rely on an entrained decision function, e.g. it can be applied to a set consisting of only two PFMs, where estimation of clustering indexes or empirical thresholds may be difficult or error prone. The proposed classifier offers an intuitive, probabilistic quantity to assess the similarity of two motifs and to decide whether they present common or distinct binding specificities. Hence, the obtained results motivate exploration of other machine learning methods to the problem of motif classification and clustering. As another practical advantage, all the motif clustering methods developed in this work automatically determined the number of clusters. Nevertheless, we see room for improvement, particularly with regard to the treatment of spacers (gaps) or different numbers of half-sites. These issues may be addressed by corresponding alignment algorithms as well as alternative IC formulations, possibly in combination with a hierarchical classification of motifs.

Motif clustering predicted between 125 and 197 motif families for vertebrate transcription factors from 35 motif classes. The smaller numbers, 125 or 135, were obtained with motif network analysis. In comparison to each other, motif network analysis revealed PFM clusters on a broader scope, whereas m2match sometimes split these further into narrower subsets. For a manually revised motif classification our results suggest an arrangement on three levels. Below the class level, motif families as defined here represent distinct specificities, e.g. different half-sites. The third level (motif subfamilies) can group more specific arrangements. The treatment of heterogeneous complexes remains to be determined for now. In addition, one could allocate classes into superclasses following the classification for TF proteins [20]. Our study has provided a good foundation of data sets and tools to work towards a honed motif classification. A possible application is the study of similar specificities across transcription factor classes, which can lead to further insights regarding interactions or interference of signaling pathways or other regulatory systems e.g. in host-pathogen interactions. Combined with a classification of transcription factor proteins, a motif classification can also support prioritization of poorly characterized TF subfamilies for experimental investigation of their binding properties. A next goal is to make the computational methods available as freely accessible web tools for applications outlined in the beginning.

## Materials and Methods

### Classification of Transfac PFMs for vertebrate transcription factors

This study used a set of 1001 PFMs from the Transfac database [2] version 2011.3, which we classified into 35 classes on the basis of DNA-binding domain annotations and manual revision. The classes, their sizes, as well as the assigned matrices are listed in Table S8. All of the motif classes correspond to distinct types of protein DNA-binding domains with the exception of the *GENINI* class, which contains initiator motifs. For ETS, IRF, and MYB as well as for FORKHEAD and RFX families (Table S8) we digressed from the protein classification [20] by focusing on the narrower family level instead of the transcription factor class level.

We manually assigned matrices of transcription factors having multiple domains with a DNA-binding property to a single *binding* class. All matrices of TFs with both HOX and POU domains were added to the HOX class. The HOX/ZFC2H2 motifs V$AREB6_01, V$AREB6_02, V$AREB6_03, V$AREB6_04, V$DELTAEF1_01 were classified as ZFC2H2 motifs, because Ikeda and Kawakami have shown in the respective study that DNA-binding specificities of AREB6 (ZEB1) are mainly determined by the zinc finger domains [29]. Further, we added all PFMs corresponding to TFs with a PAX domain or to TFs with both PAX and HOX domains to the HOX class in order to investigate the similarity between these motifs and other HOX PFMs (see Results). Finally, several Transfac motifs were associated with factors containing both a BZIP and a ZFC2H2 domain, e.g. V$CREBP1CJUN_01, V$CREBP1_Q2, V$CREBP1_01, V$CREBATF_Q6. Since to our knowledge the zinc finger domain in these proteins does not contribute directly to DNA-binding, these matrices were treated together with other BZIP matrices.

### Jaspar CORE database

A second set of PFMs was obtained from the Jaspar CORE database version 2009. Since the redundant and non-redundant matrix libraries differed in size by only 17 entries, we used the redundant set of 476 motifs for the assessment of motif comparison methods. For motif clustering we compiled the set of 71 non-zinc finger matrices following previous studies, where the matrix Athb-1 was replaced by the one named ATHB-5 (MA0110.1). The data set is listed in Dataset S1. Computational experiments carried out with Jaspar used the Jaspar families and matrix assignments.

### Local ungapped motif alignment

Our program m2match implements a local ungapped alignment algorithm for DNA sequence motifs. Here we compare motifs described by the standard Position-specific Frequency Matrix model, a 4×L matrix whose elements are the frequencies or probabilities of individual nucleotides in each of position (column). The algorithm searches for the best (highest scoring) alignment consisting of at least *min(5,L_x_,L_y_)* consecutive columns in each of two motifs *x* and *y* with lengths *L_x_* and *L_y_*, respectively. The score of the alignment of two PFMs is determined by the sum of aligned column scores. For this study we implemented the column-wise scoring methods listed in Table 3.

**Table 3:** Column-wise scores implemented in m2match.

Column-wise score	Formula
Euclidean distance (ED)	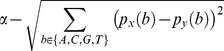
Sum-of-squared distances (SSD)	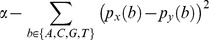
Pearson correlation coefficient (PCC)	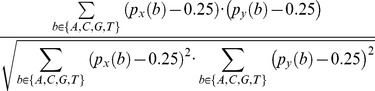
Log-sum-of-odds (LSO)	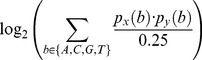
LSO with Kullback-Leibler divergence (LSO.KL)	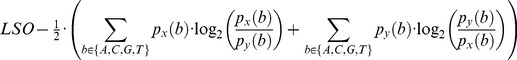

In Table 3, p_x_(b) and p_y_(b) are the nucleotide probability distributions in aligned columns of matrices *x* and *y*. Probabilities were estimated on the basis of raw PFM entries. We denote as raw PFM the model which is stored in a database and typically requires further transformation in order to be used for binding site prediction or motif comparison. A pseudo-count of 1 was added to counts of nucleotide occurrences. If a raw PFM did not contain nucleotide counts we set the normalized frequencies so that a minimal value >0 and not less than 10^−3^ was guaranteed. Like Mahony et al. [6] we took care to remove uninformative flanking positions. Here we trimmed positions for which the highest difference between any two nucleotide frequencies was less than .25. The selection of α parameters used by ED and SSD scores is described below.

The Euclidean distance score was previously applied by Gupta et al. in the Tomtom tool [12]. Further, the SSD and Pearson correlation coefficient (PCC) metrics were analyzed in detail by Mahony et al. [6], whereas the SSD metric for motif comparison was introduced in [7]. The Log-sum-of-odds (LSO) score has been successfully applied to comparison of HMMs modeling protein alignments [16]. Its extension by the Kullback-Leibler (KL) divergence has been used to characterize conserved non-coding sequence motifs [17].

### Information coverage

We extended ED and SSD alignment scores (sums of individual ED or SSD column scores) with the information coverage. The IC quantifies the proportion of the information contained in both aligned PFMs which is covered by the alignment. The information *I(p_x_)* of a PFM column was defined in terms of the entropy as:
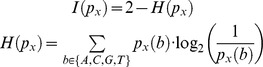
We calculated the information coverage as follows:
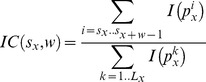
where s_x_ is the start position of the alignment in matrix *x* and *w* is the width of the ungapped alignment. Here, we added indices i and k for corresponding columns within the entire motif. To combine the information coverage with ED and SSD scores we defined the following extended scores:
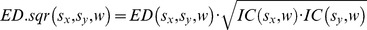


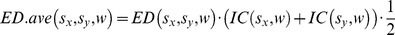


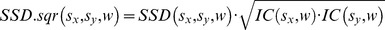


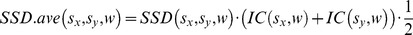
where 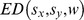 and 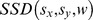 denote the sum of ED and SSD scores for the alignment with start points *s_x_* and *s_y_* in matrix *x* and *y* as well as width *w*, respectively. Both *sqr* and *ave* extensions multiply the total alignment by a value in the interval [0,1]. Hence, the *IC* moves the raw score towards 0 the less information of the motifs is covered by the alignment.

### Evaluation of motif comparison methods by “best hit” and “class-depth” statistics

Motif comparison methods were evaluated on both the set of classified Transfac PFMs as well as the Jaspar CORE data set (see above). Following previous studies the *best hit* statistic provides for a way to assess the ability of a method to identify members of the same TF class for an uncharacterized input motif [6]. A leave-one-out test is performed where each motif is removed and compared to all remaining motifs in the database. One then records which proportion of held-out motifs matched a pattern from the same transcription factor class as best hit. Since our Transfac data set was considerably larger than the one used in [6] and contained many similar motifs, we in addition calculated the *class-depth* statistic. We have developed this statistic in order to record for each held-out motif which proportion of PFMs from the same class can be detected before the first false positive. Since this approach yields several proportion values for each class, we calculated robust statistics consisting of upper and lower quartiles as well as the median.

Aside from a list of column-wise score methods the comparison included Mosta [13] and KFV [14] as third-party tools. Mosta was invoked with two GC contents of 40% (the program default, here denoted as Mosta.GC.4) and of 50% (denoted as Mosta.GC.5). Column-wise scores were implemented in m2match and encompassed LSO, LSO.KL, ED, SSD, and PCC scores. Both SSD and PCC scores are also available in the STAMP tool [9].

Note that matrices from the family named *Other* in the Jaspar CORE data set, which gathers potentially unrelated motifs, were considered in determining false positive matches, but PFMs from that family were not used as hold-out set.

### Optimization of α parameters for ED and SSD scores

**Figure 6:**
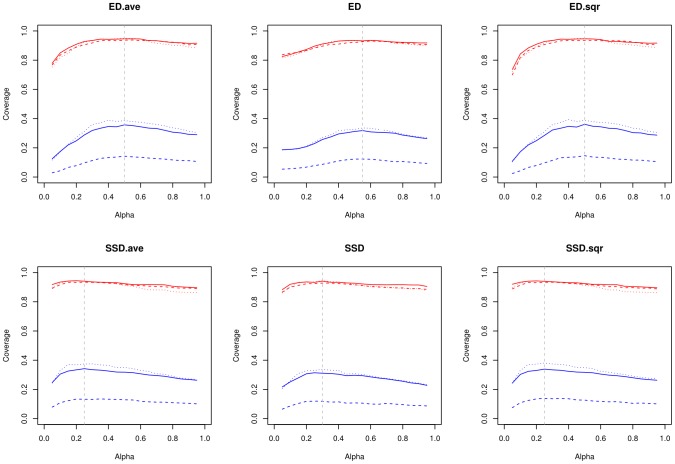
Optimization of α-parameters applied in ED and SSD scores. Optimization selected α- parameters for best performance according to best hit (red) and class-depth statistics (blue) in the range from 0.05 to 0.95. Different subsets of TF classes such as the 5 largest (dashed lines), classes with at least 20 (solid lines) as well as with at least 10 matrices (dotted lines) were also considered. Optimal alpha values were 0.5 for ED.ave and ED.sqr scores, 0.55 for the ED score, 0.25 for SSD.ave and SSD.sqr scores, as well as 0.3 for the SSD score and are indicated by gray dotted lines.

We determined α parameters for ED, ED.sqr, ED.ave, as well as SSD, SSD.sqr, and SSD.ave scores that were optimal with respect to best hit and class-depth statistics obtained on the Transfac PFM set. The results are illustrated in Figure 6. The graph for each method shows best hit (red lines) and class-depth (blue lines) statistics over a range of α values. We also considered different subsets of TF classes, which were the 5 largest classes only (dashed lines), classes with at least 20 motifs (solid lines) and classes with at least 10 motifs (dotted lines). According to our assessment, optimal alpha values were 0.5 for ED.ave and ED.sqr scores, 0.55 for the ED score, 0.25 for SSD.ave and SSD.sqr scores, as well as 0.3 for the SSD score (gray dotted lines, Fig. 6). These values were kept for all subsequent analyses.

### Construction of motif networks and extraction of clusters

Motif networks were constructed for each class of the Transfac data set. Further analysis focused on motif classes with at least 5 PFMs. In the networks each motif was connected to all other motifs which were detected with a higher score than the first non-class member. The Markov Cluster algorithm (MCL) [23] was then applied to extract clusters from motif networks. The program was used with default values. All edge weights were equal, so that the algorithm clustered motifs on the basis of the graph topological properties of the motif network. Network visualizations were created with the help of yED [30]. Alignments of transcription factor DNA-binding domains represented by at least one classified motif were compiled in [22]. Phylogenetic trees were calculated using Tree-Puzzle [31].

### Comparison of network and hierarchical cluster results

Network and m2match clusters were compared on the basis of the Rand index [32]. For two clusterings U and V over a set of N items the Rand index RI is defined as
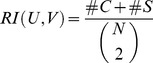
where #C is the number of item pairs in a common cluster and #S is the number of item pairs in different clusters in both clusterings. RI is a quantity in the [0,1]-interval and equals 1 for perfect agreement between two clusterings of the same set of items.

## Supporting Information

Dataset S1 71 non-zinc finger matrices from the Jaspar CORE database version 2009.
(TXT)

Figure S1 A–D) DNA-binding domain phylogenies for HMG, MADS, SMAD and STAT proteins. E) Clustering of STAT motifs by m2match with subgroups corresponding to matrices with different half-site numbers.
(PDF)

Figure S2 Network visualization of HOX motif clusters.
(TIF)

Figure S3 Network visualization for ZFC2H2 motif clusters.
(TIF)

Figure S4 Motif clustering results by m2match for the classes ETS, FORKHEAD, BHLH, BZIP and ZFC4-NR.
(DOC)

Table S1 Summary of motif clustering results using motif network analysis or the hierarchical clustering approach implemented in m2match with ED.sqr or ED scores.
(XLS)

Table S2 Motif networks constructed using ED.sqr scores and clusters extracted by MCL for classes with zero or one MCL cluster.
(DOC)

Table S3 Motif networks constructed using ED.sqr scores and clusters extracted by MCL for classes with two MCL clusters.
(DOC)

Table S4 Motif families extracted according to motif networks and MCL clusters.
(XLS)

Table S5 Rand indexes comparing motif network-based and m2match clusters.
(XLS)

Table S6 Clusters proposed by m2match for HOX motifs.
(XLS)

Table S7 Clusters proposed by m2match for ZFC2H2 motifs.
(XLS)

Table S8 Motif classes assigned to 1001 Transfac motifs.
(XLS)

Text S1 Motif network clusters and DNA-binding domain phylogenies for the classes BHLH, BZIP, HMG, MADS, REL, SMAD and STAT as well as of clusters obtained for HOX and ZFC2H2 motifs.
(DOC)

Text S2 Listing of HOX motif clusters. Each row corresponds to one cluster. “V$”-prefixes were omitted.
(TXT)

Text S3 Listing of ZFC2H2 motif clusters.
(TXT)
