# Supplementary material for: A Discriminative Approach for Unsupervised Clustering of DNA Sequence Motifs
Source: PLoS Comput Biol. 2013 Mar 21;9(3):e1002958. doi: 10.1371/journal.pcbi.1002958 (PMC3605052; doi:10.1371/journal.pcbi.1002958)

A-D. DNA-binding phylogenies for HMG, MADS, SMAD and STAT classes. Phylogenies were compiled as described in Material and Methods. Colors highlight protein subgroups corresponding to motif clusters.
E. Clustering of Transfac motifs calculated with m2match

A. HMG class

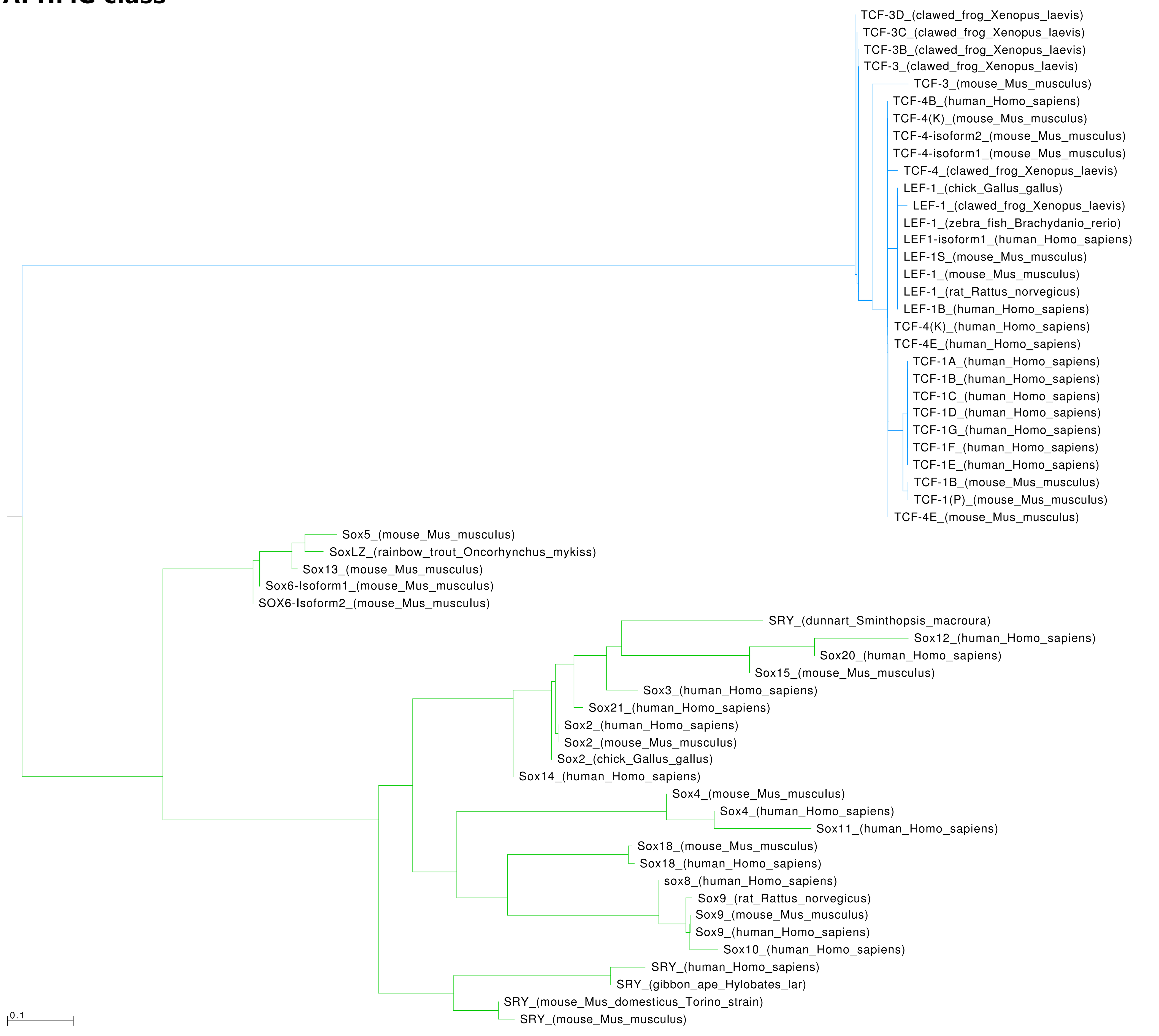

B. MADS class

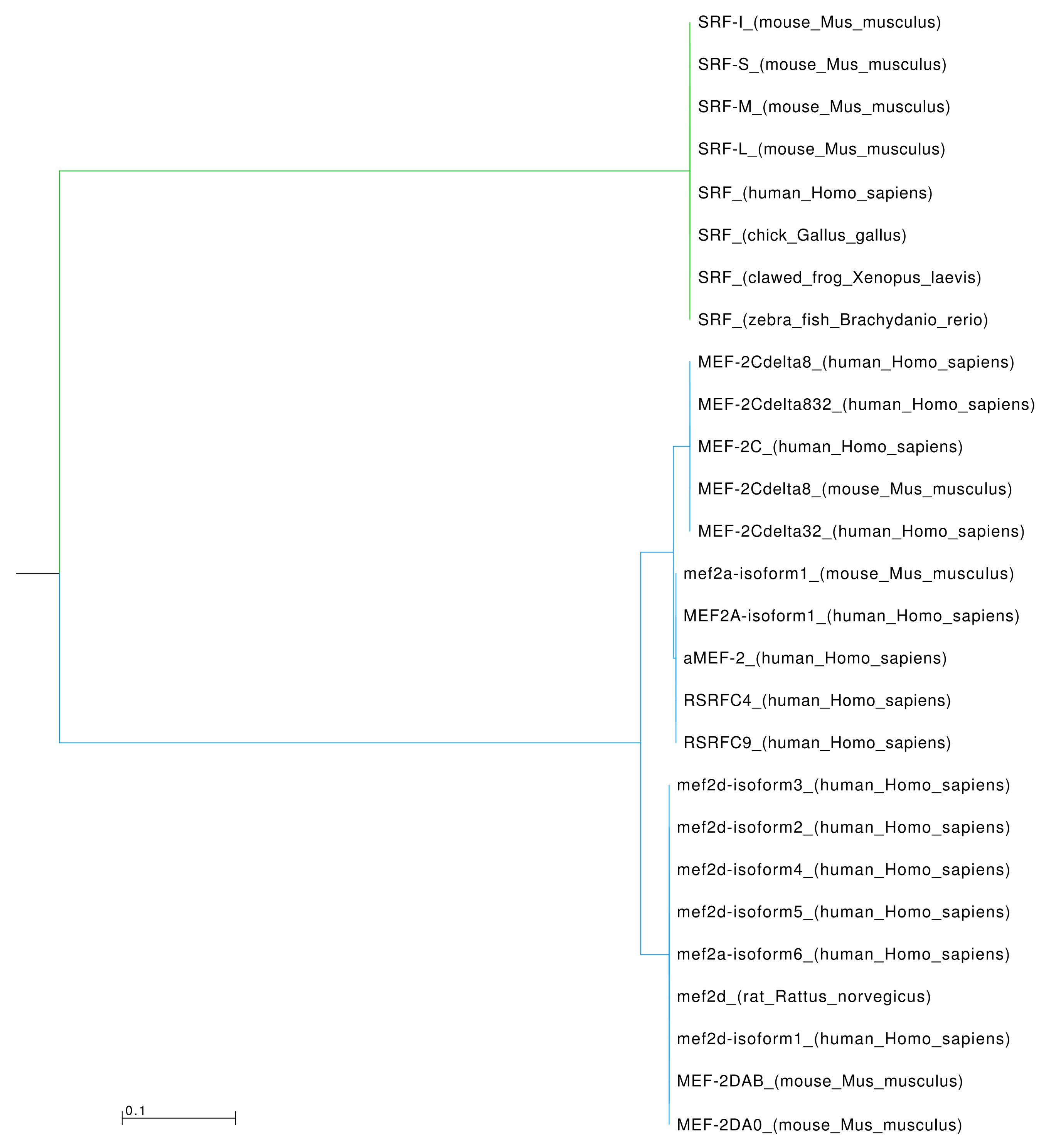

C. SMAD class

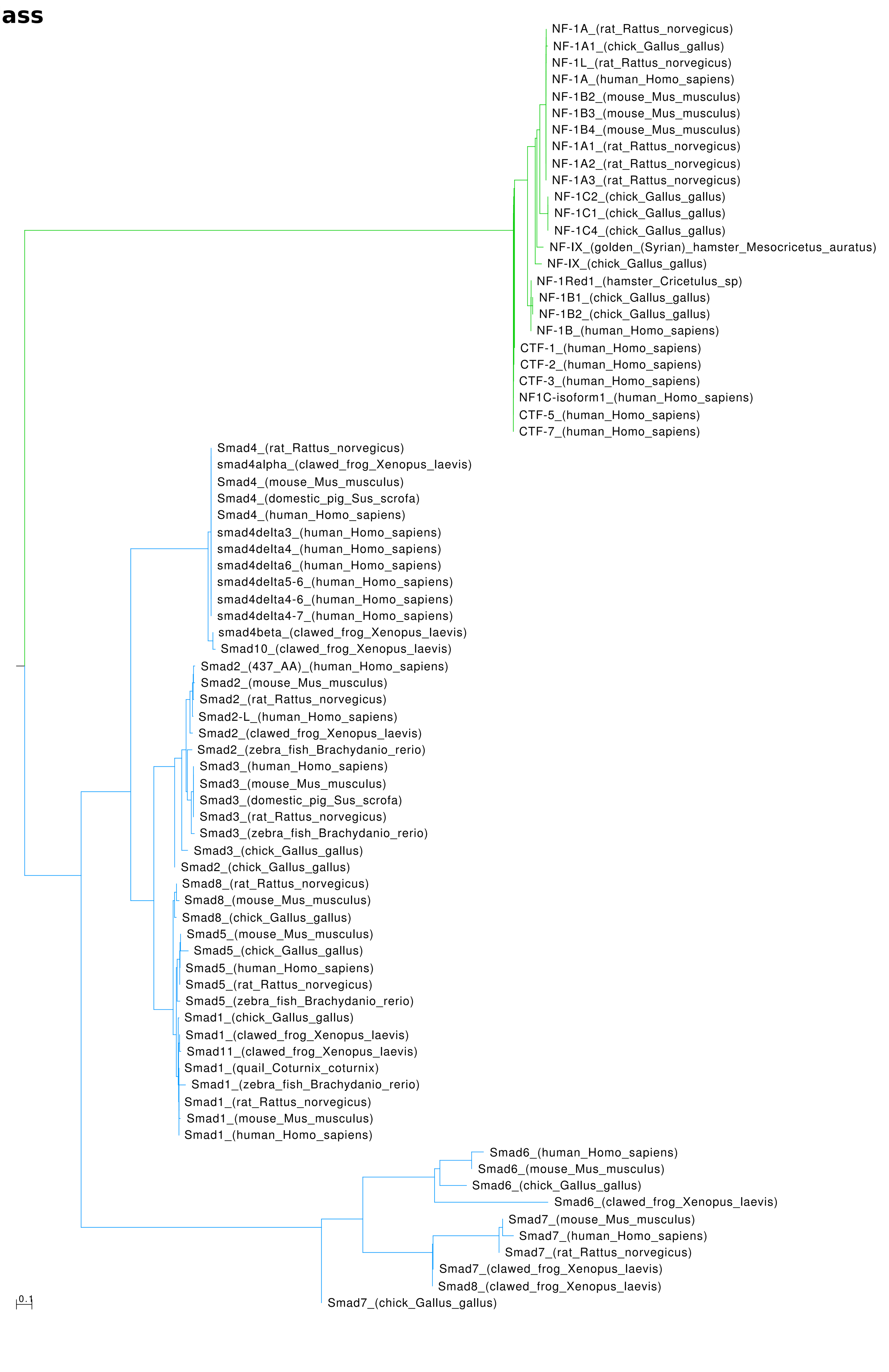

D. STAT class

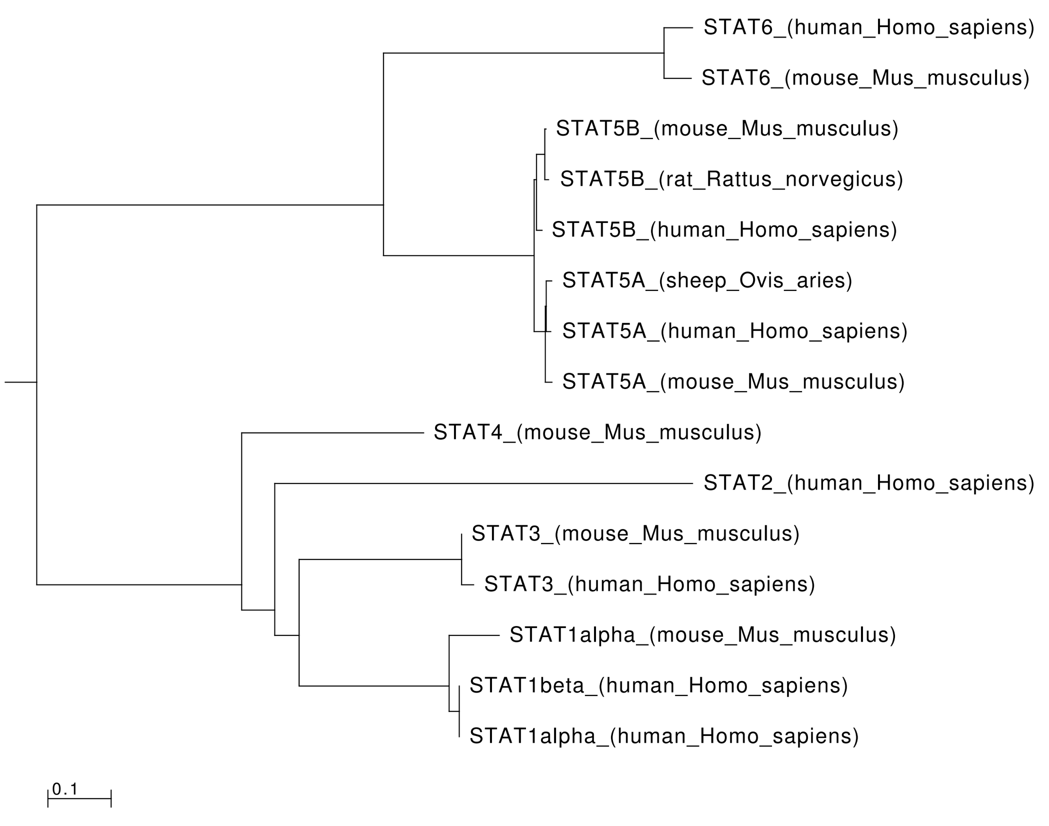

E. m2match clustering of Transfac STAT motifs

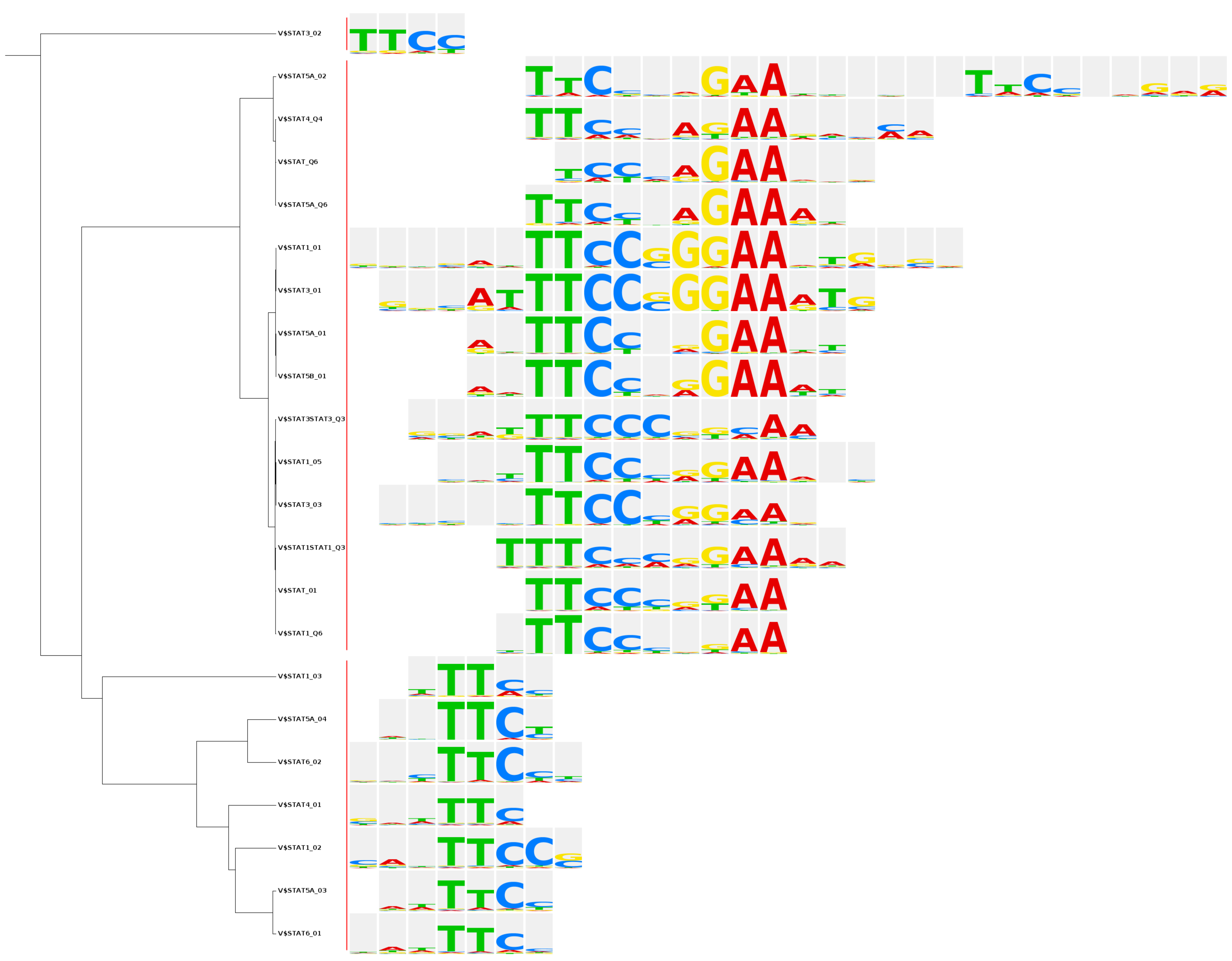

Supplement: Figure S1 — A–D) DNA-binding domain phylogenies for HMG, MADS, SMAD and STAT proteins. E) Clustering of STAT motifs by m2match with subgroups corresponding to matrices with different half-site numbers. (PDF) [file pcbi.1002958.s002.pdf]
