# Supplementary figures and images for: A Discriminative Approach for Unsupervised Clustering of DNA Sequence Motifs
Source: PLoS Comput Biol. 2013 Mar 21;9(3):e1002958. doi: 10.1371/journal.pcbi.1002958 (PMC3605052; doi:10.1371/journal.pcbi.1002958)

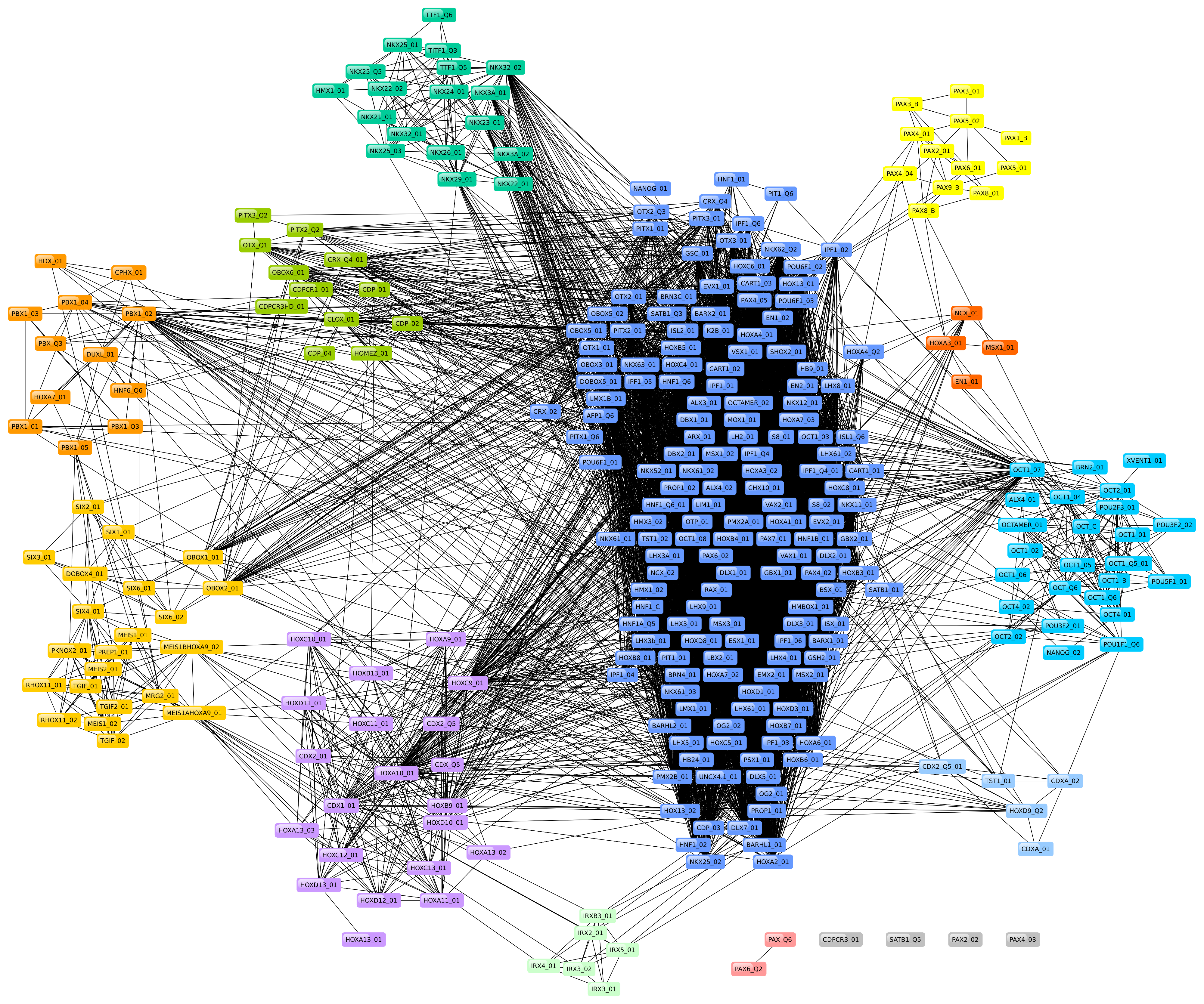

Supplement: Figure S2 — Network visualization of HOX motif clusters. (TIF) [file pcbi.1002958.s003.tif]

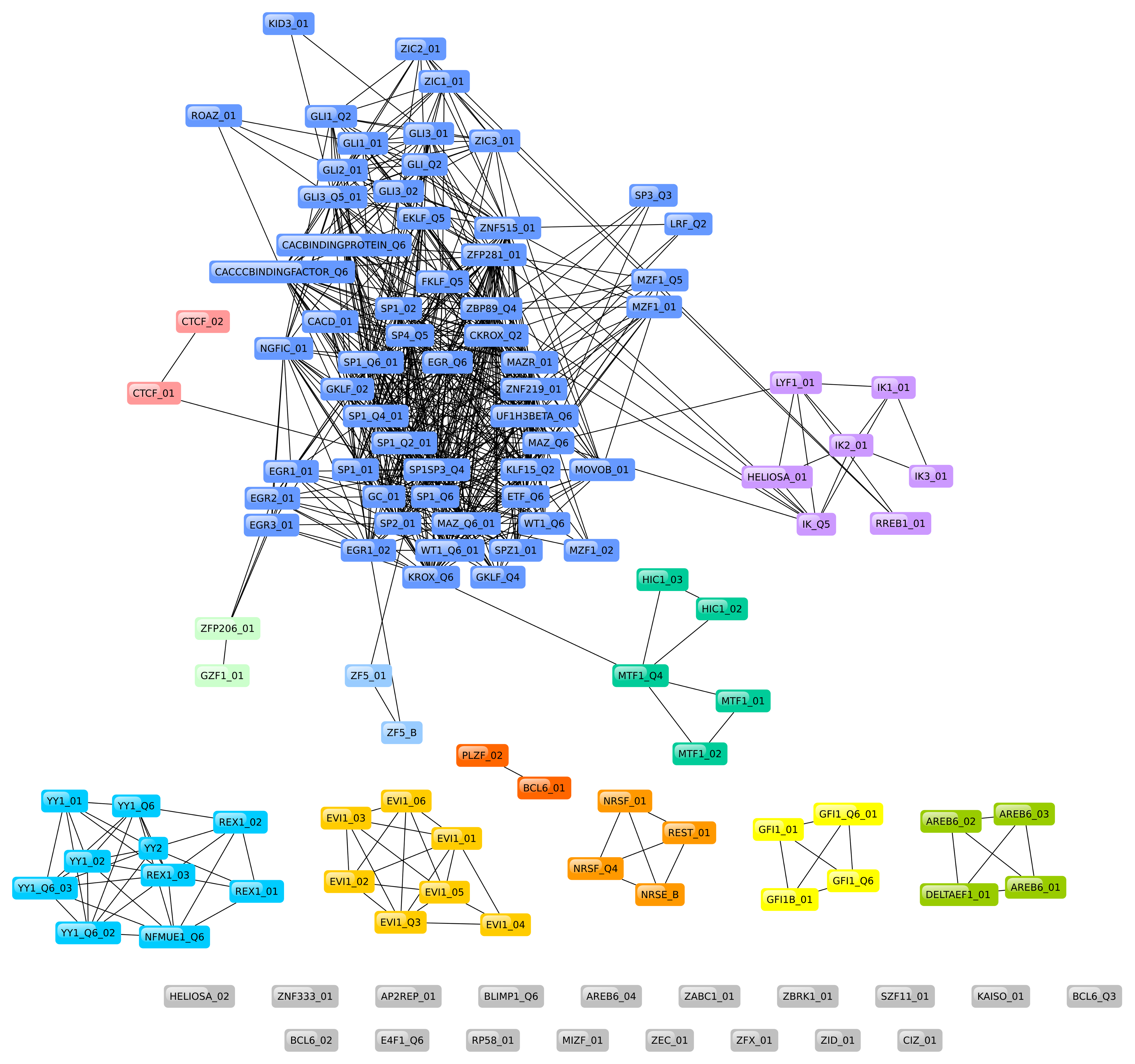

Supplement: Figure S3 — Network visualization for ZFC2H2 motif clusters. (TIF) [file pcbi.1002958.s004.tif]
