# Supplementary material for: A Discriminative Approach for Unsupervised Clustering of DNA Sequence Motifs
Source: PLoS Comput Biol. 2013 Mar 21;9(3):e1002958. doi: 10.1371/journal.pcbi.1002958 (PMC3605052; doi:10.1371/journal.pcbi.1002958)

**S4**

In this document we present networks of motifs highlighting the clusters as computed using our method m2match. Nodes with common colors were assigned to the same motif cluster. These figures can be compared to respective visualizations of clusters extracted by the network-based approach.

The last page depicts the hierarchical clustering result for the nuclear receptor (ZFC4-NR) class using m2match. Motif clusters are indicated by red lines.

**ETS class**


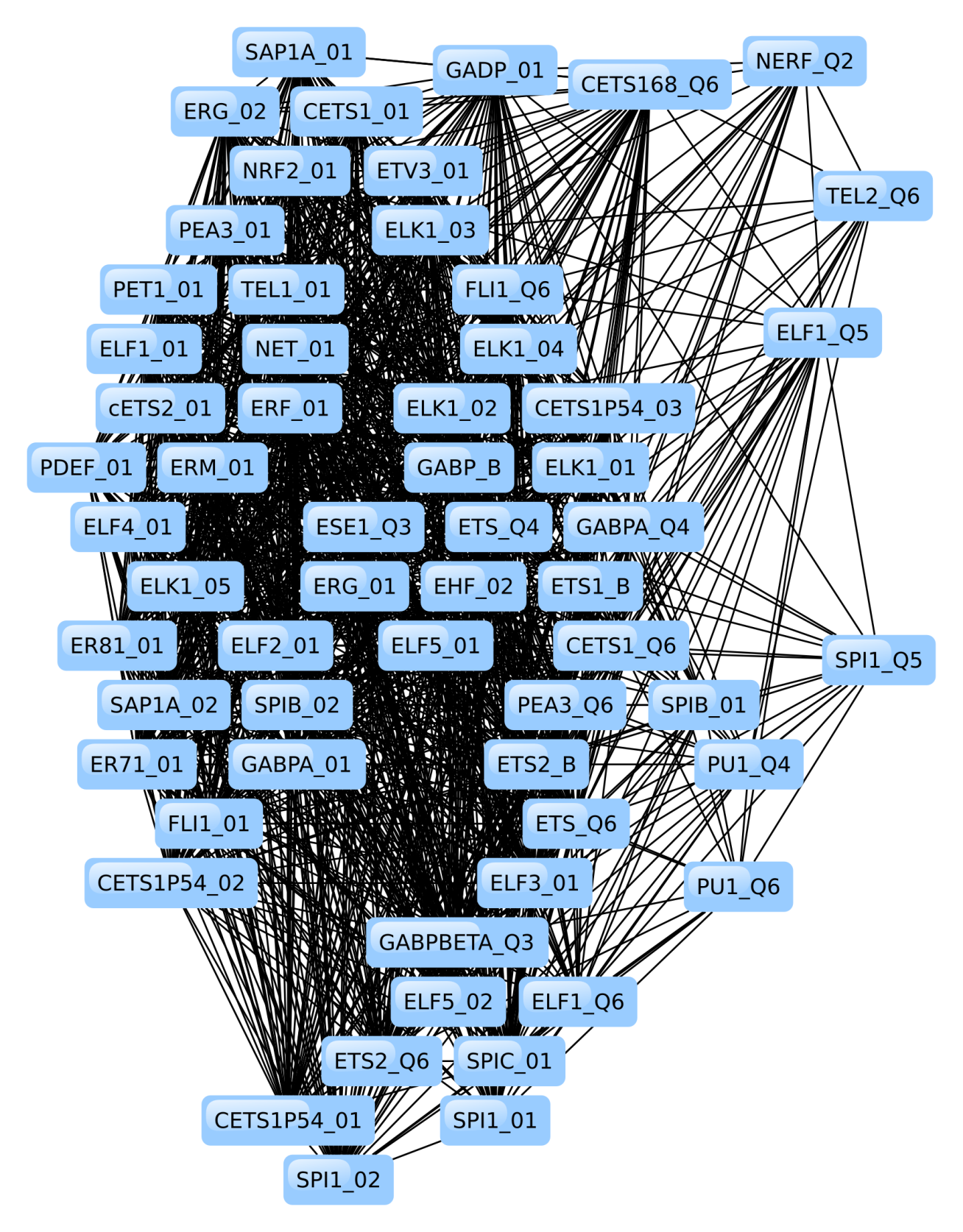


**Forkhead class**


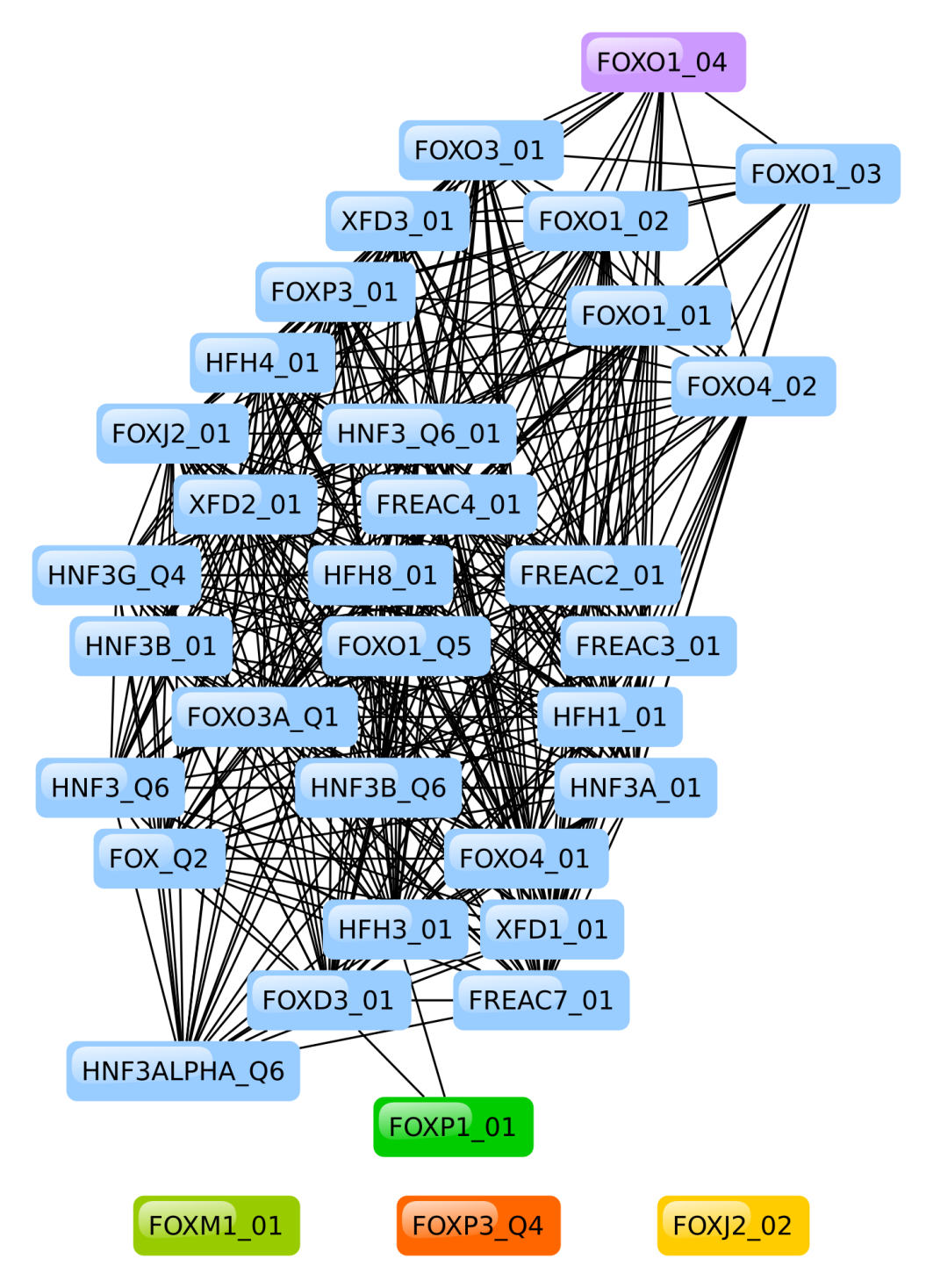


**BHLH class**


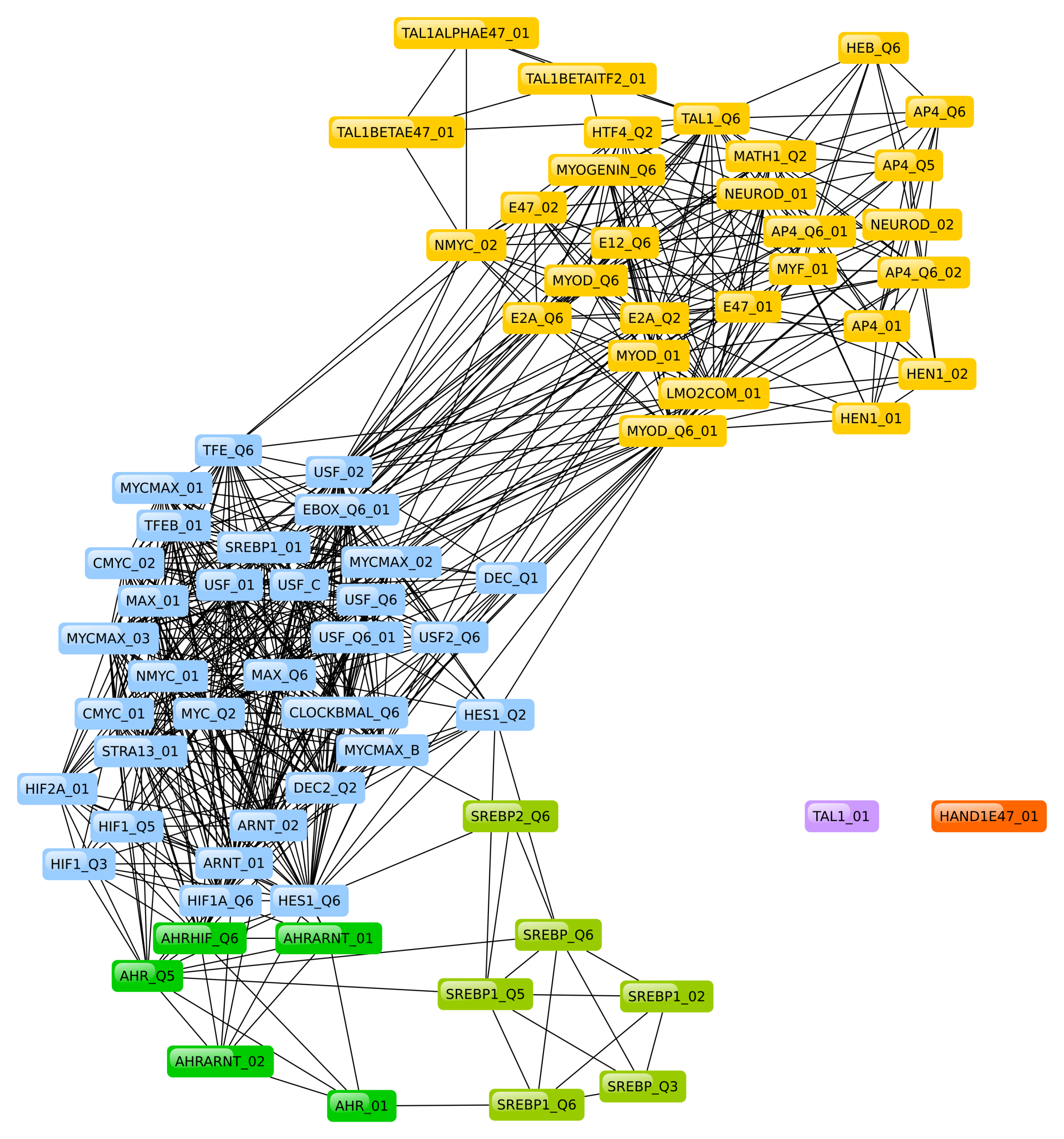


**BZIP class**


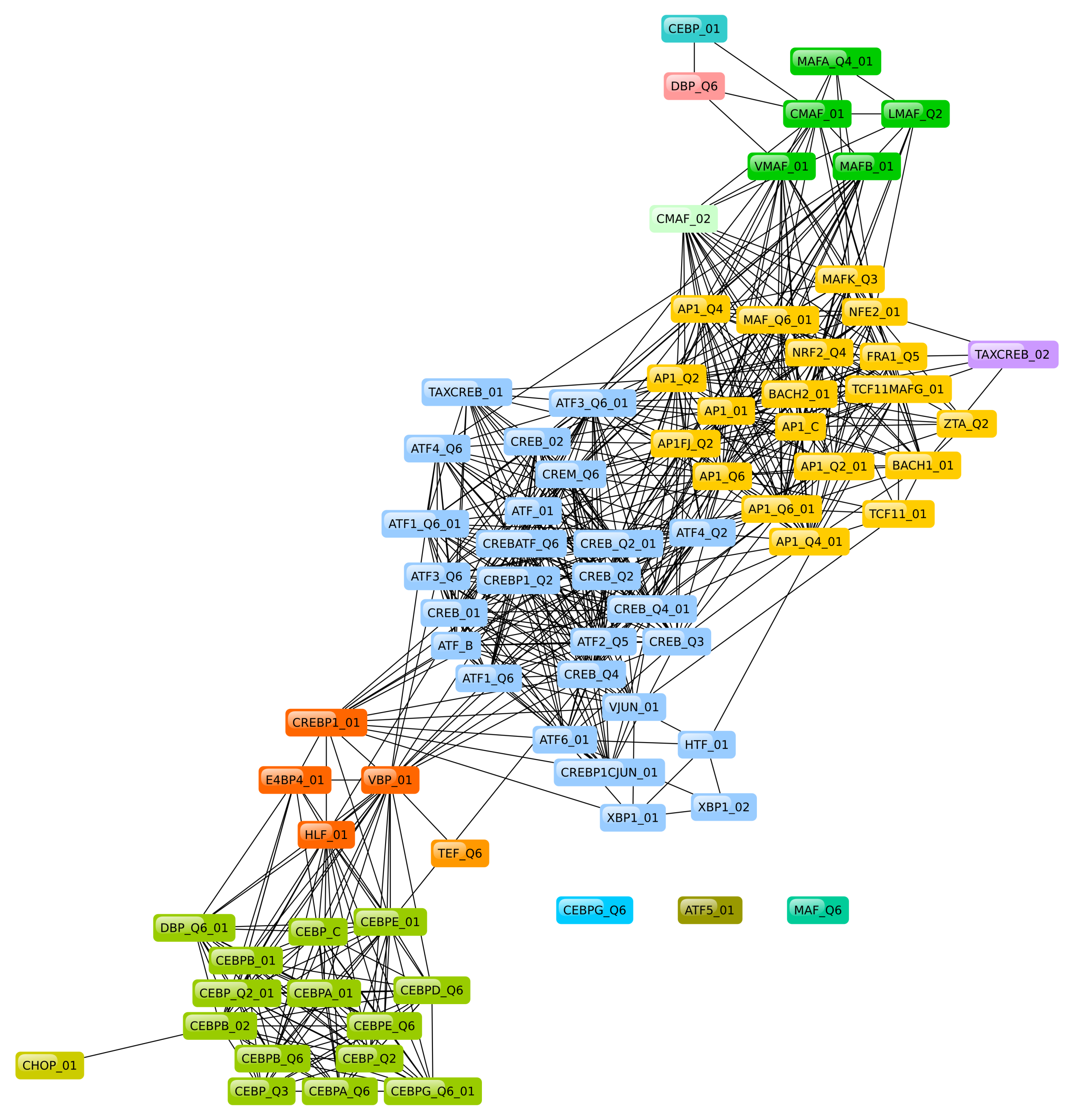


**ZFC4-NR class**


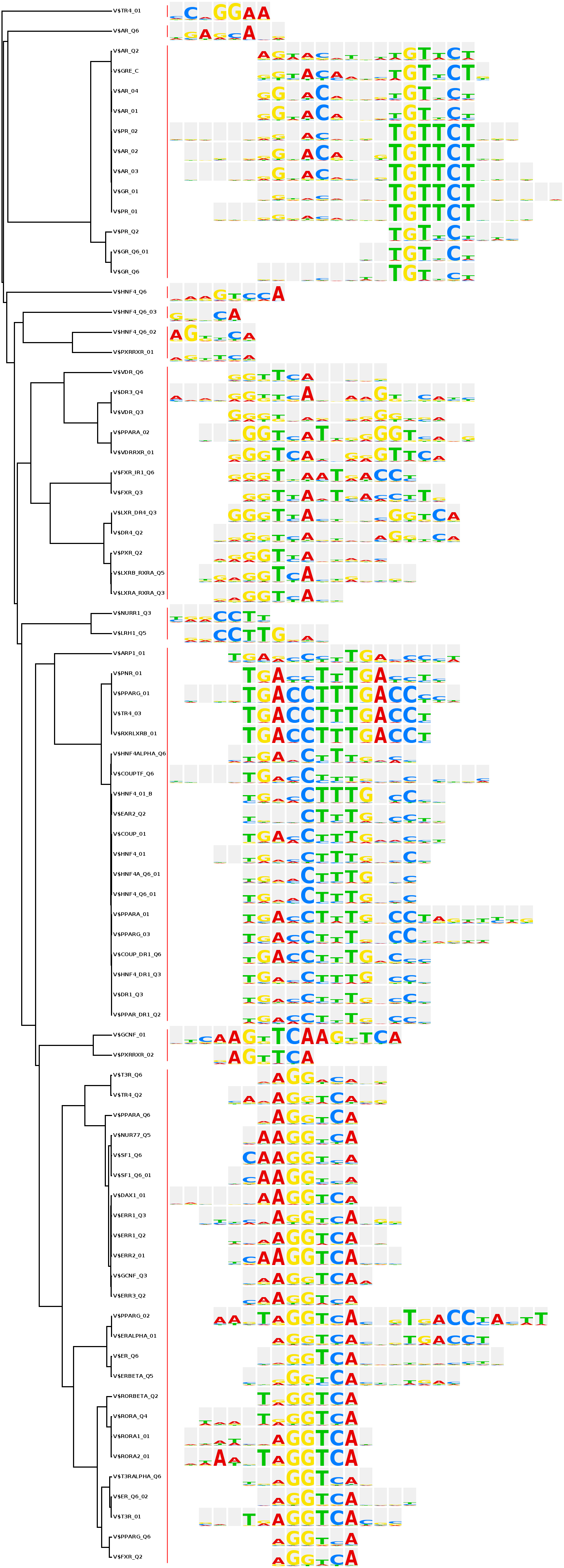

Supplement: Figure S4 — Motif clustering results by m2match for the classes ETS, FORKHEAD, BHLH, BZIP and ZFC4-NR. (DOC) [file pcbi.1002958.s005.doc]
