# Supplementary material for: A Discriminative Approach for Unsupervised Clustering of DNA Sequence Motifs
Source: PLoS Comput Biol. 2013 Mar 21;9(3):e1002958. doi: 10.1371/journal.pcbi.1002958 (PMC3605052; doi:10.1371/journal.pcbi.1002958)
Supplement: Table S2 — Motif networks constructed using ED.sqr scores and clusters extracted by MCL for classes with zero or one MCL cluster. (DOC) [file pcbi.1002958.s007.doc]

**Motif networks constructed using ED.sqr scores and clusters extracted by MCL**

The following table shows networks of classes with zero (ATHOOK class) or one MCL cluster. Note that we did not consider disconnected motifs (gray nodes) as cluster, although in some cases we suspect that the separation of single matrices corresponds to motif families with different DNA-binding properties, see e.g. the distinction between matrices of v- and c-Myb factors and the CDC5 motif in the MYB class (Table S3) or the separate placement of the EBF motif within the REL class (Text S1). A motif was disconnected from its class members if at least one non-class member achieved a higher score than all other class members. V$-prefixes were omitted from Transfac matrix identifiers in network illustrations as all of the nodes correspond to vertebrate transcription factors.

| **#Motifs** | **#Row** | **Transcription factor class** |
| --- | --- | --- |
| 5 | 1 | **ATHOOK**  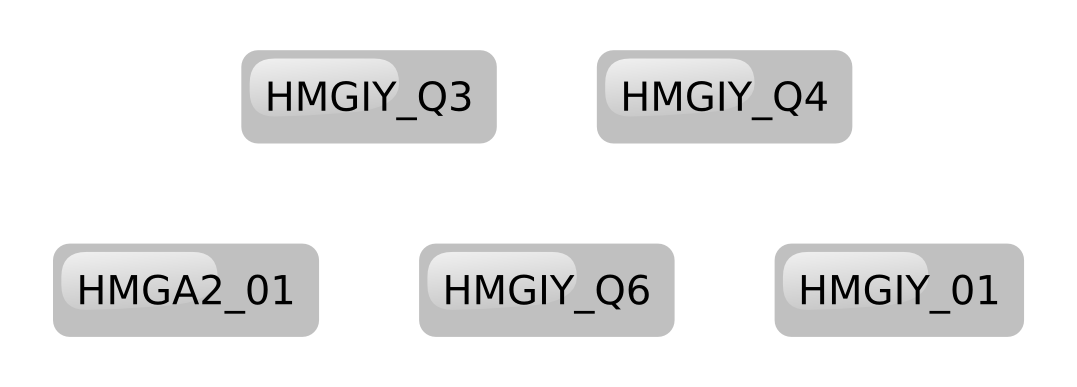 |
| 10 | 2 | **BHSH**  **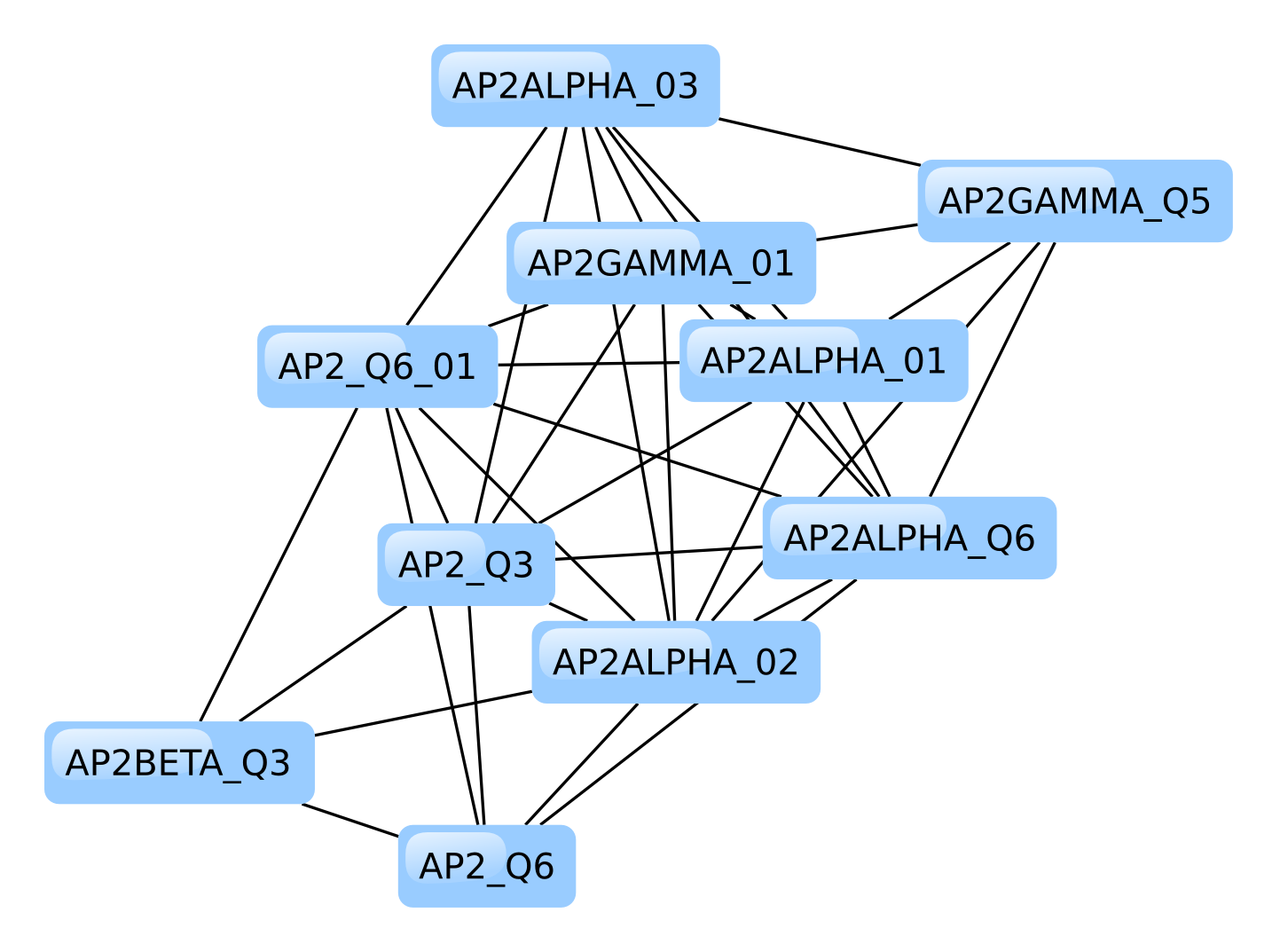** |
| 6 | 3 | **DM**  **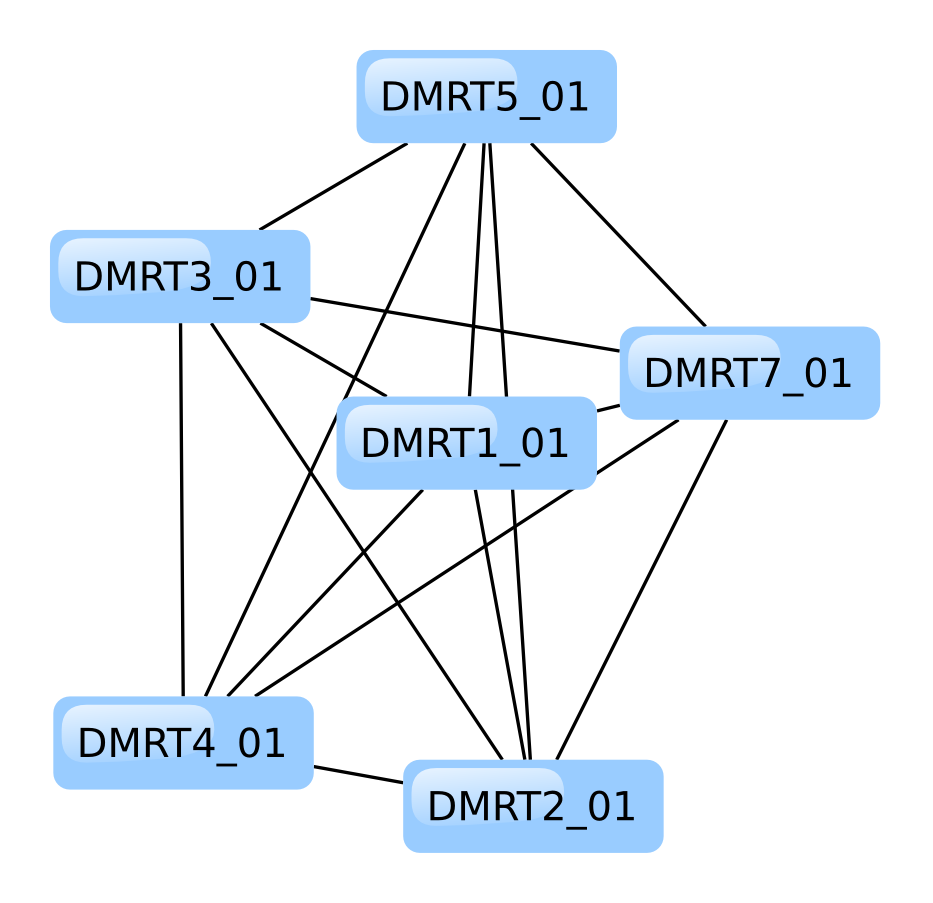** |
| 60 | 4 | **ETS**  **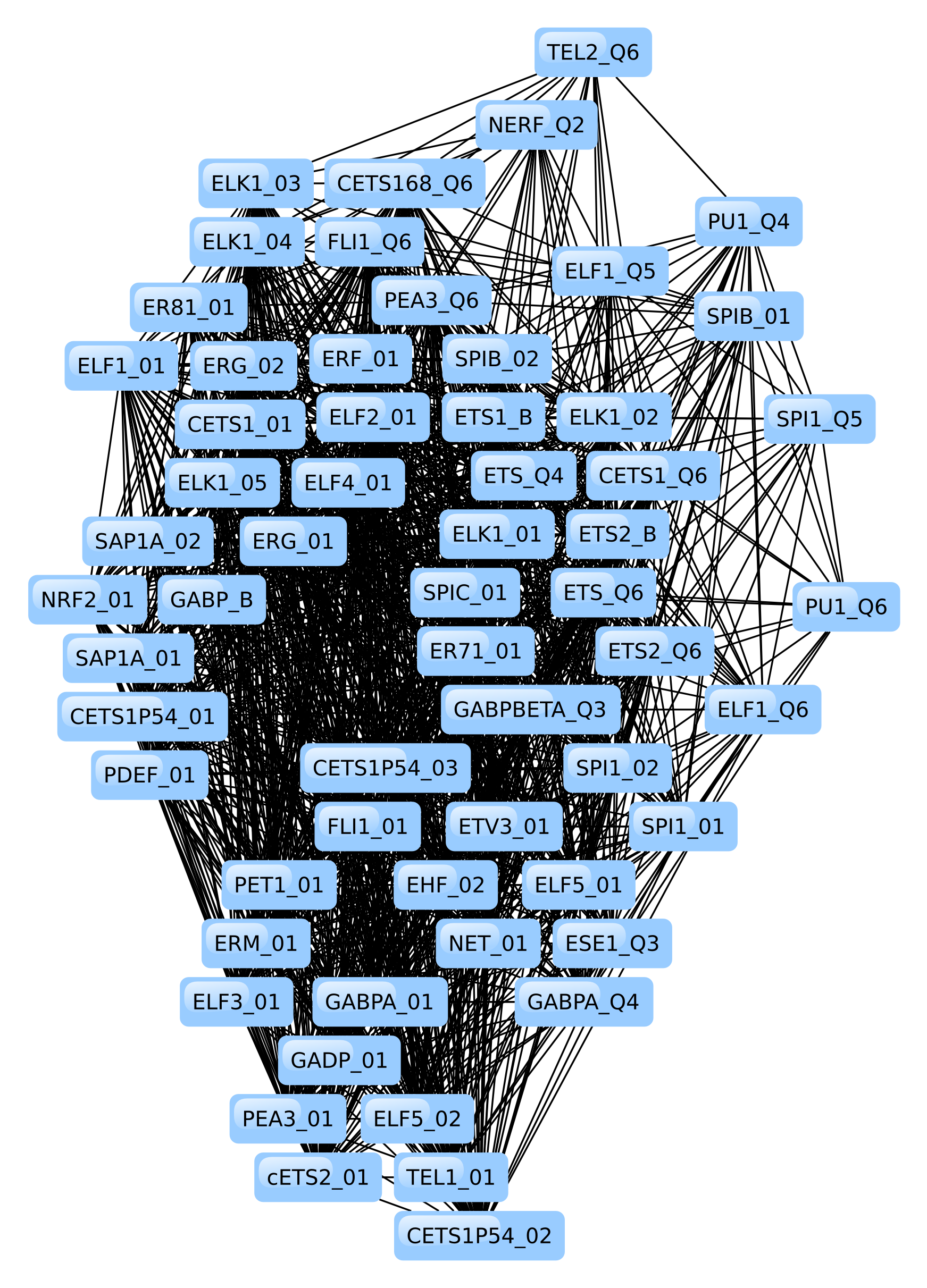** |
| 35 | 5 | **FORKHEAD**  **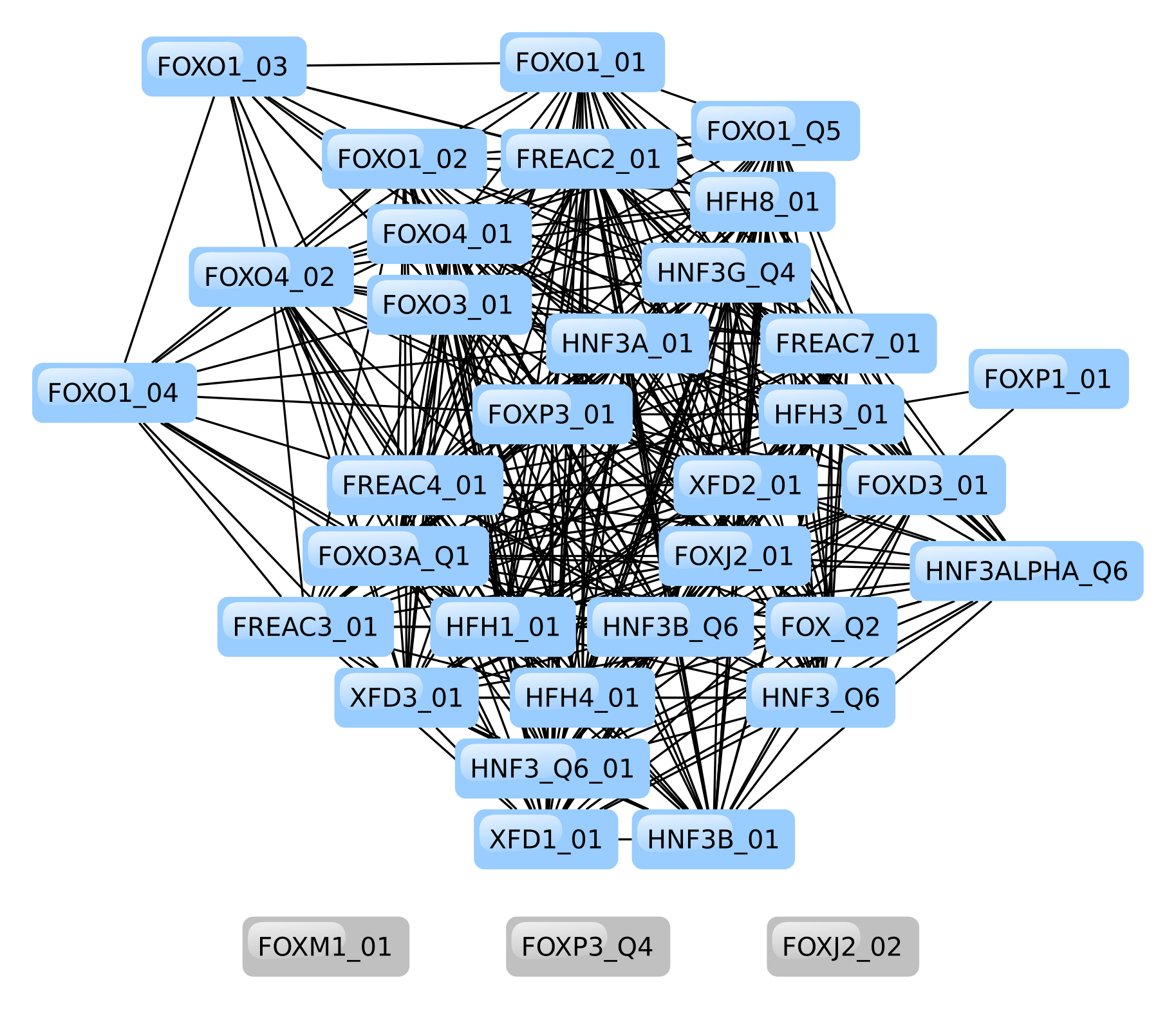** |
| 8 | 6 | **HISTONE**  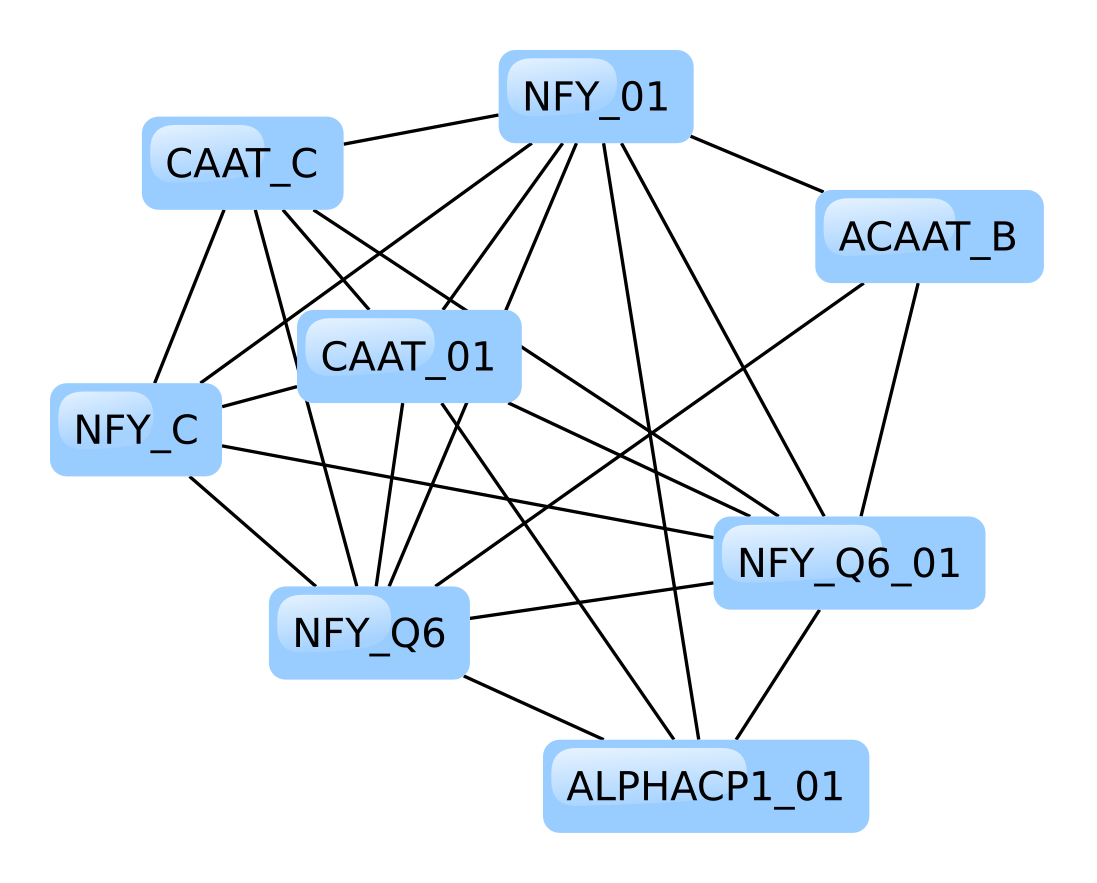 |
| 5 | 7 | **HSF**  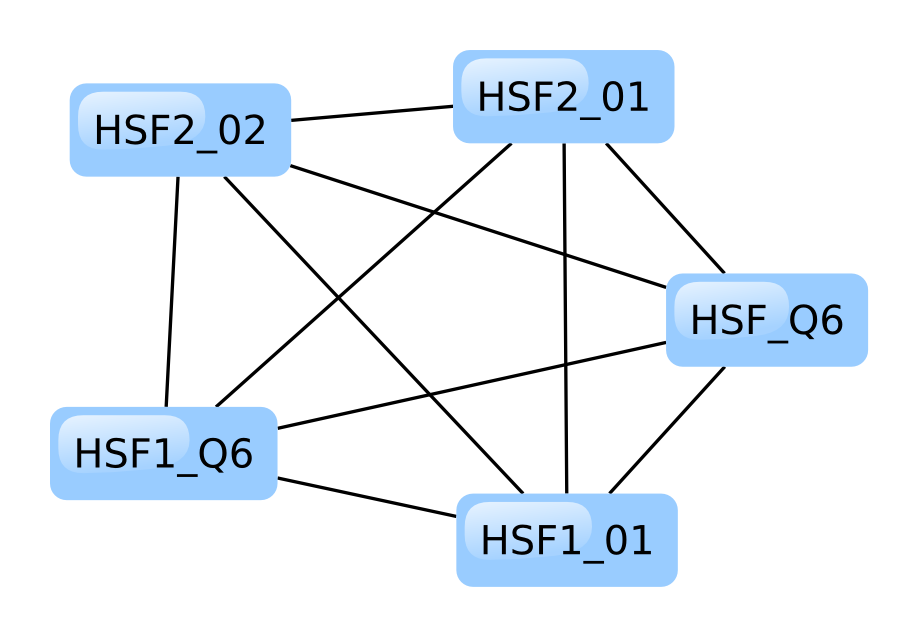 |
| 7 | 8 | **P53**  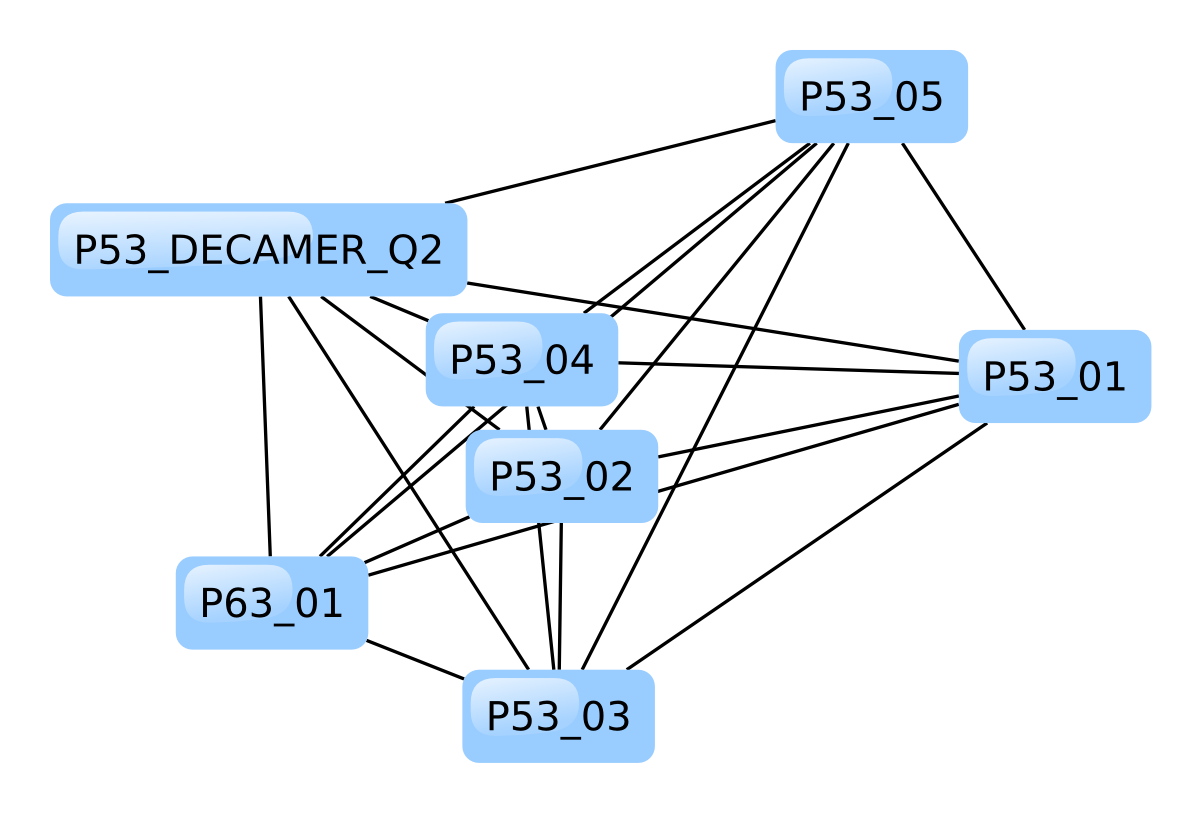 |
| 5 | 9 | **RFX**  **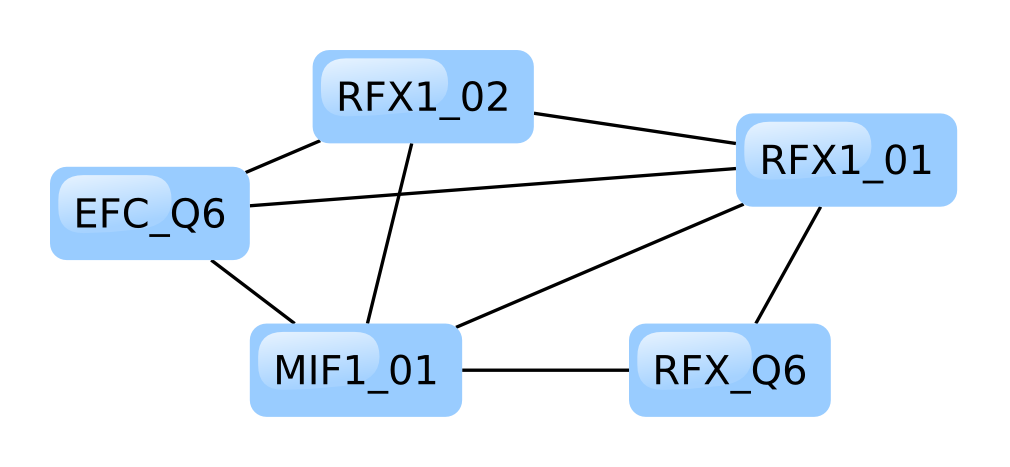** |
| 9 | 10 | **TBX**  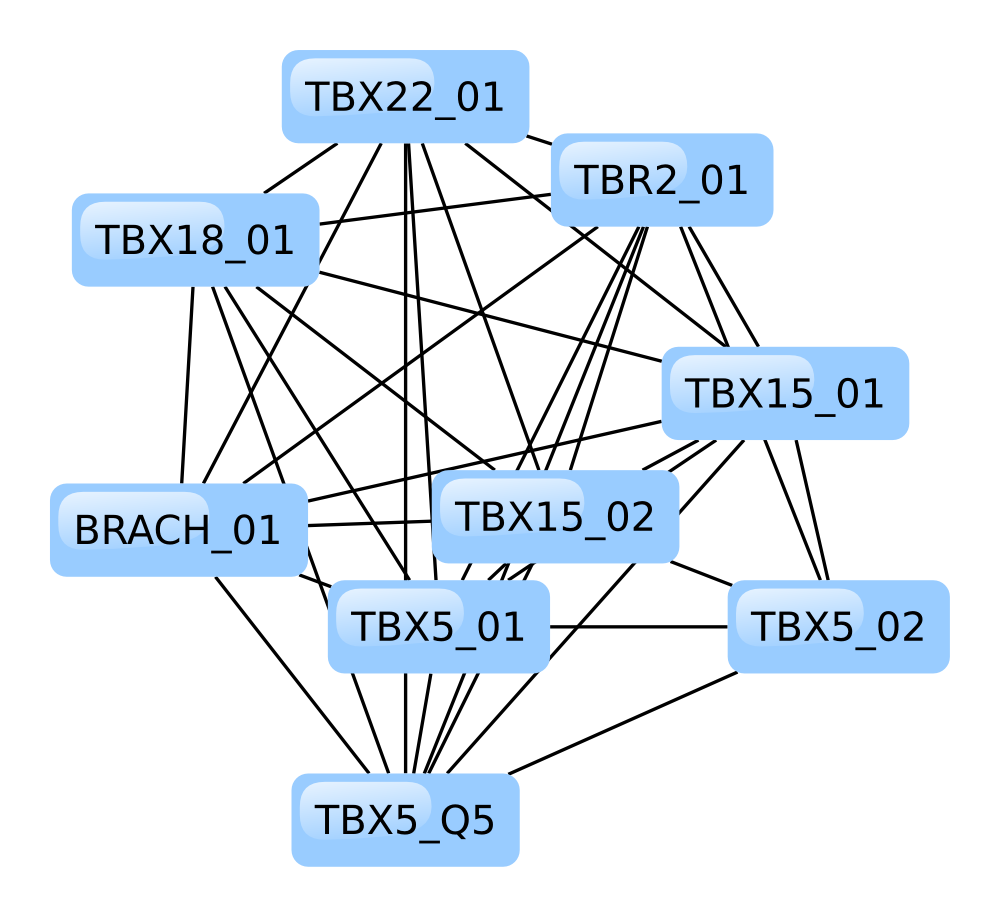 |
| 18 | 11 | **ZFGATA**  **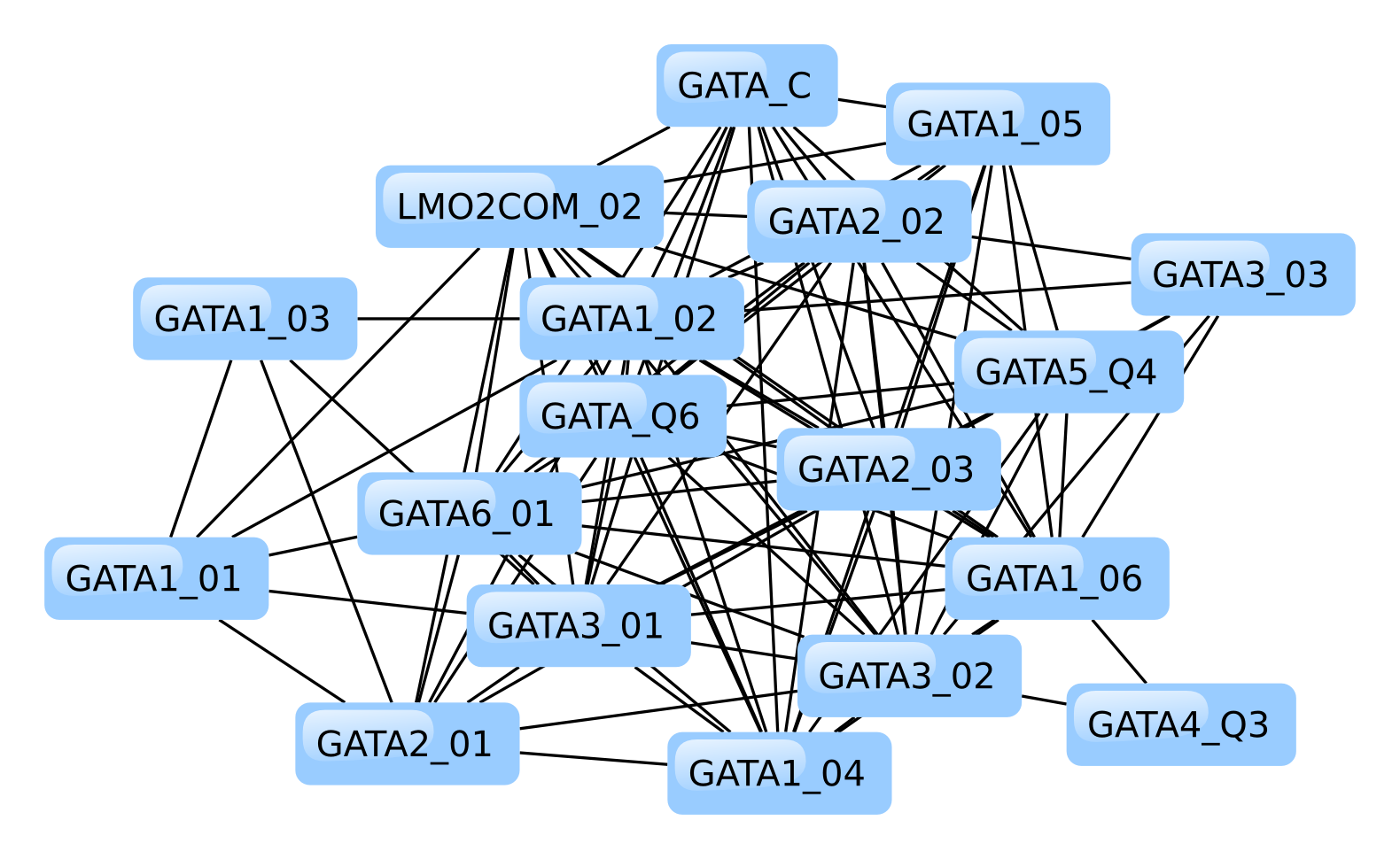** |
