# Supplementary material for: A Discriminative Approach for Unsupervised Clustering of DNA Sequence Motifs
Source: PLoS Comput Biol. 2013 Mar 21;9(3):e1002958. doi: 10.1371/journal.pcbi.1002958 (PMC3605052; doi:10.1371/journal.pcbi.1002958)
Supplement: Table S3 — Motif networks constructed using ED.sqr scores and clusters extracted by MCL for classes with two MCL clusters. (DOC) [file pcbi.1002958.s008.doc]

**Motif networks constructed using ED.sqr scores and clusters extracted by MCL**

The following table shows classes with two MCL clusters. Clusters are indicated by different node colors. See also Table S2 for further description.

| **#Motifs** | **#Row** | **Transcription factor class** |
| --- | --- | --- |
| 24 | 1 | **E2F**  **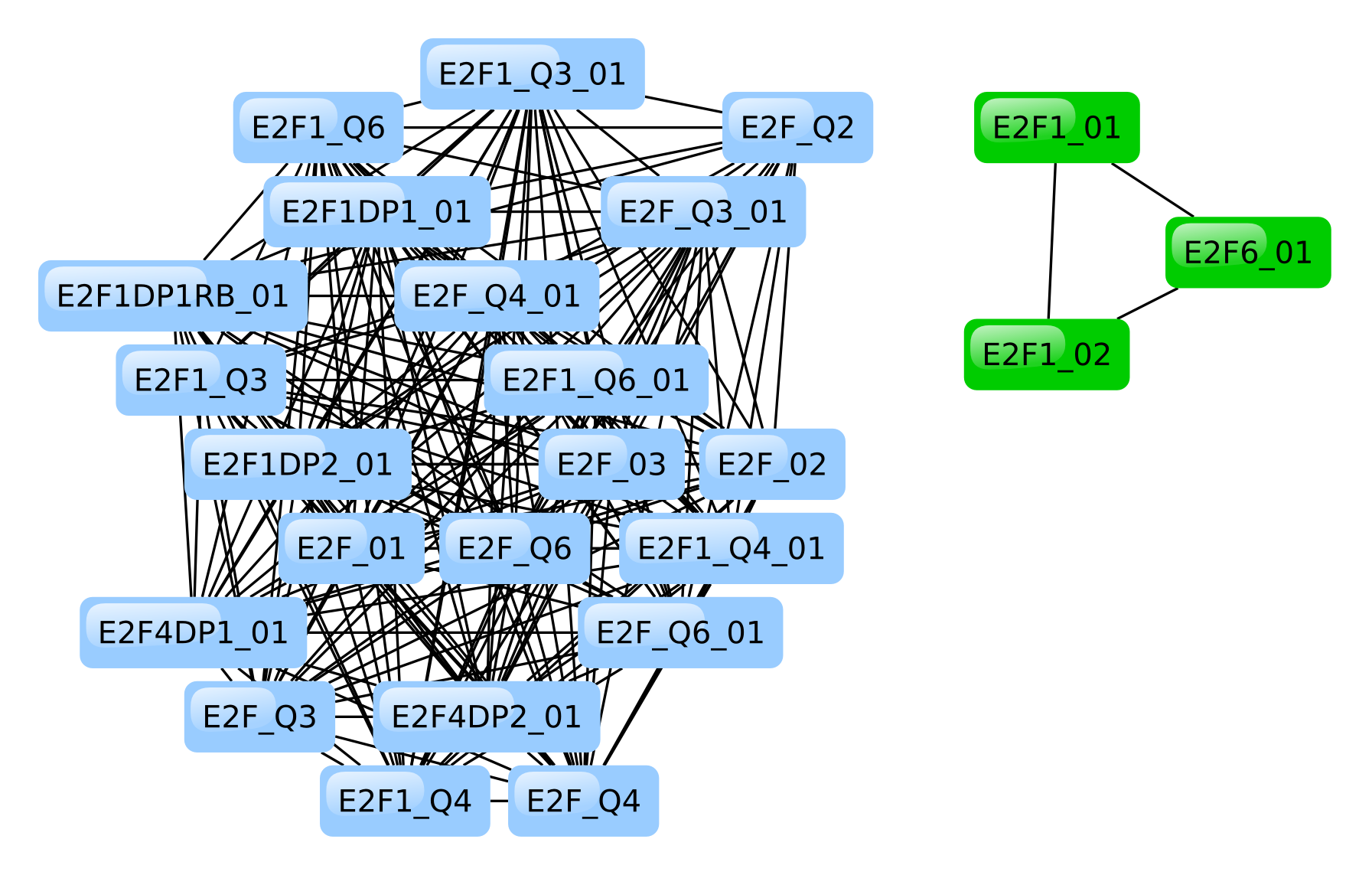** |
| 6 | 2 | **GENINI**  **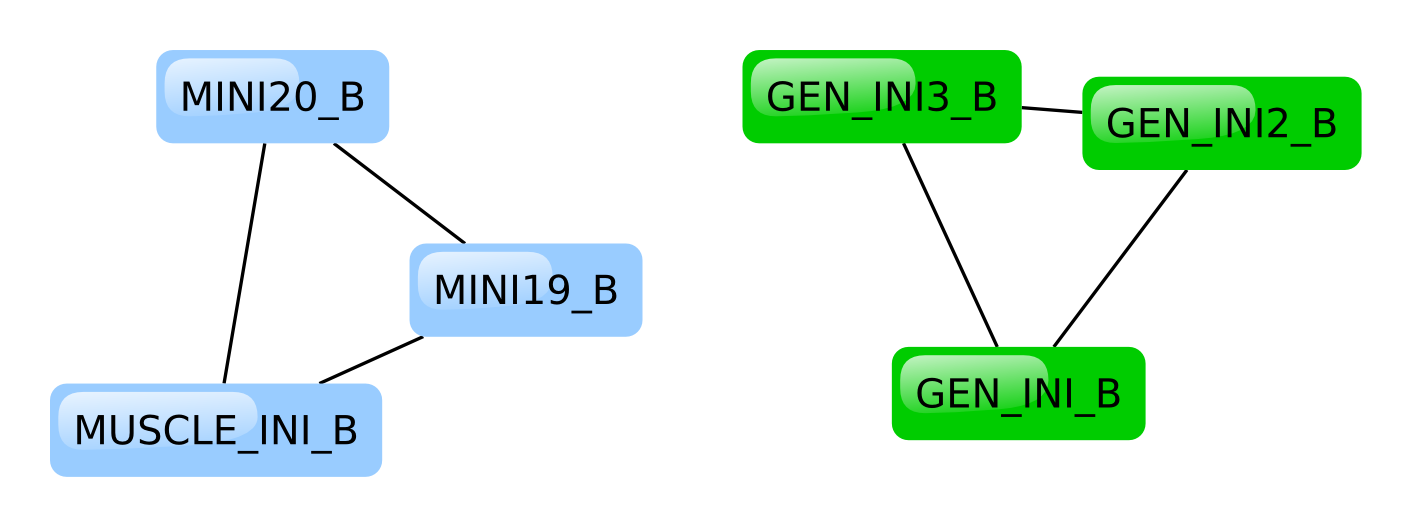** |
| 20 | 3 | **HMG**  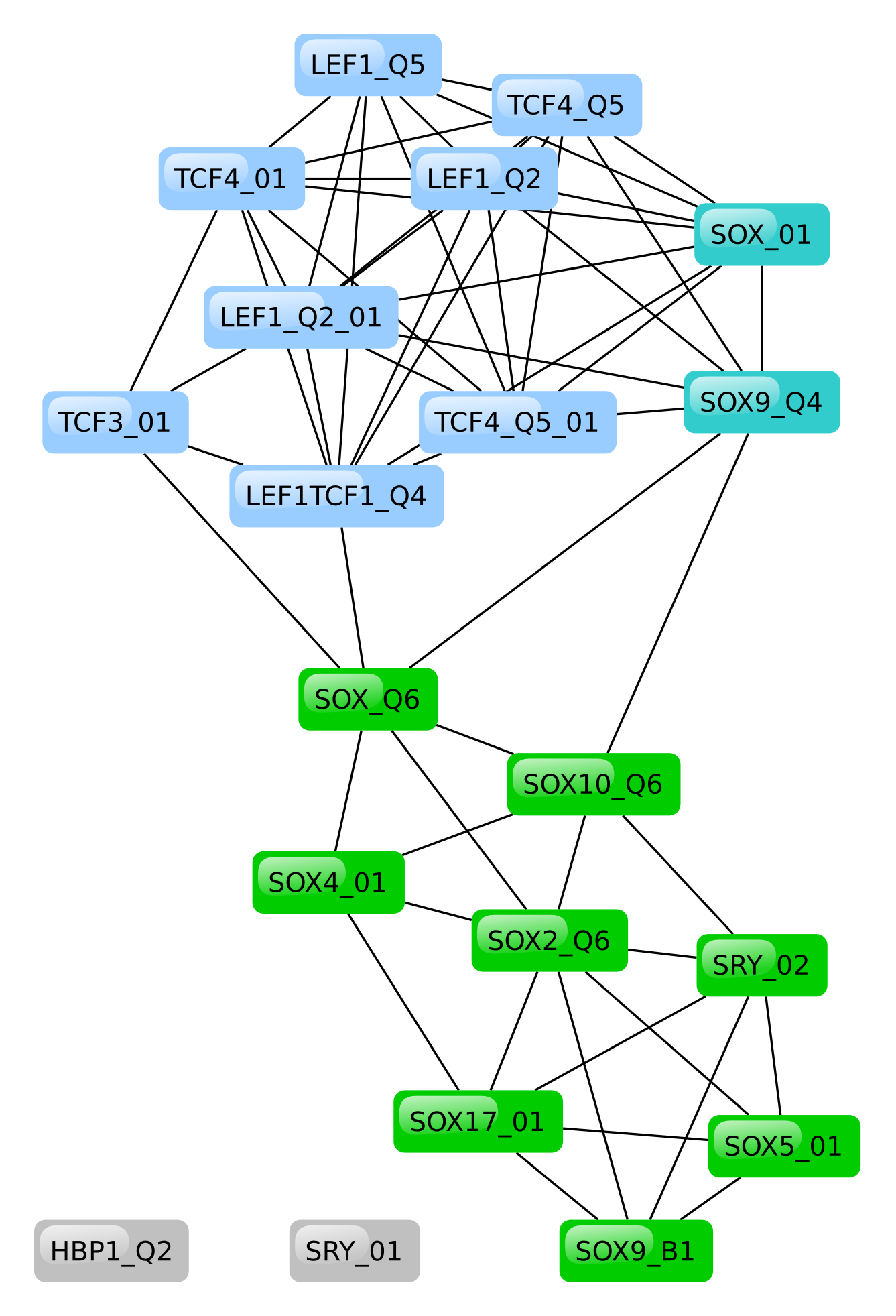 |
| 13 | 4 | **IRF**  **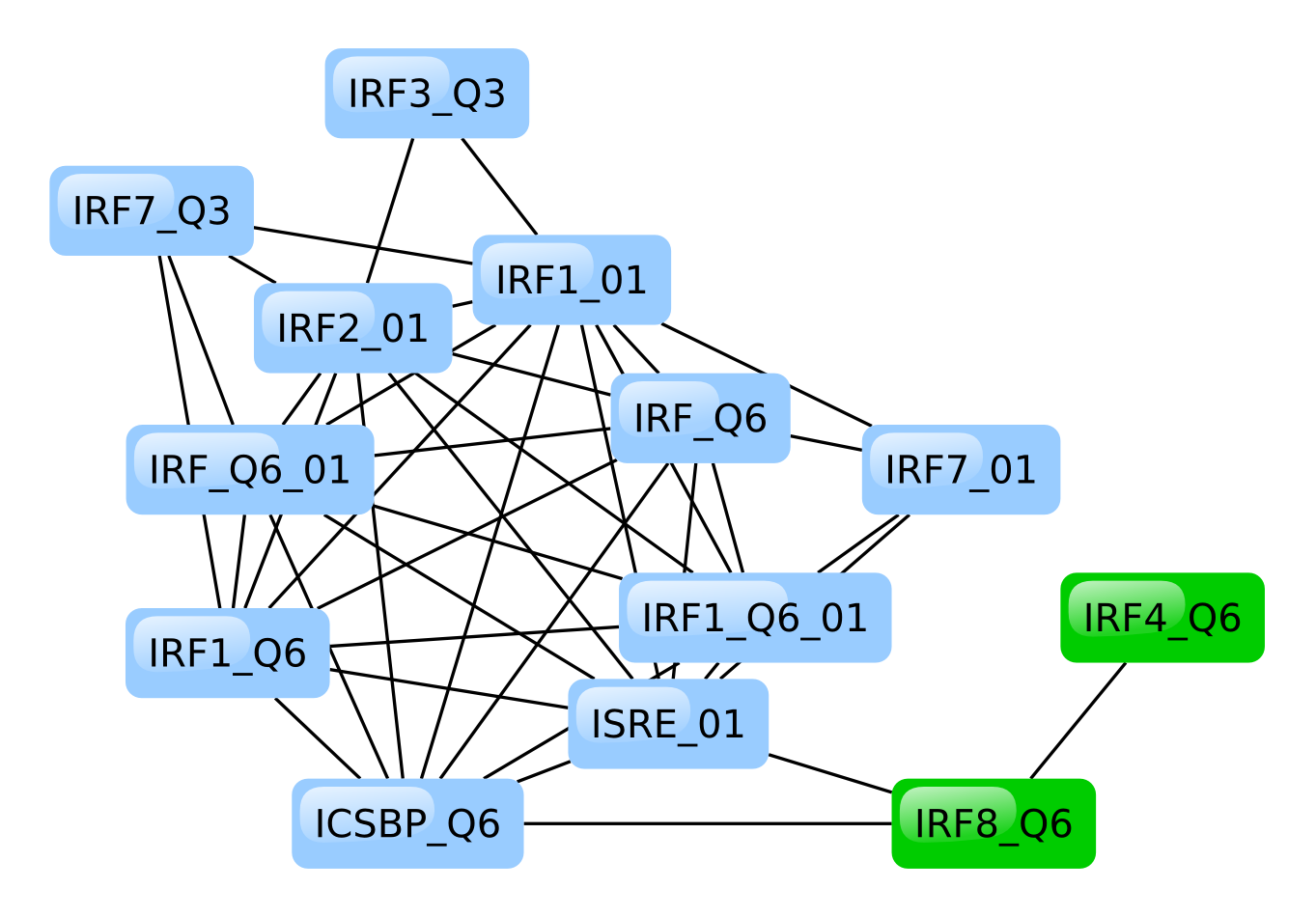** |
| 22 | 5 | **MADS**  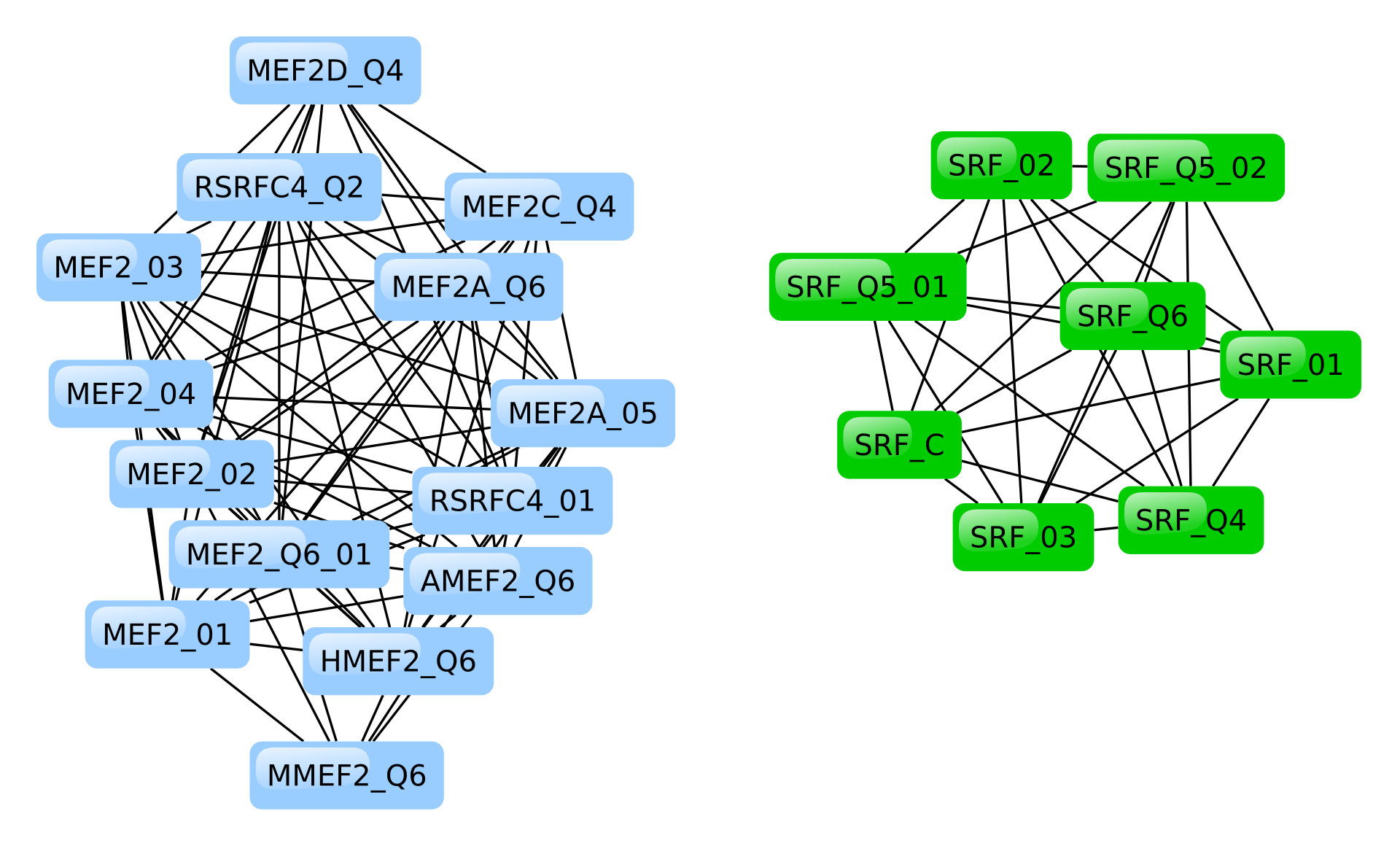 |
| 8 | 6 | **MYB**  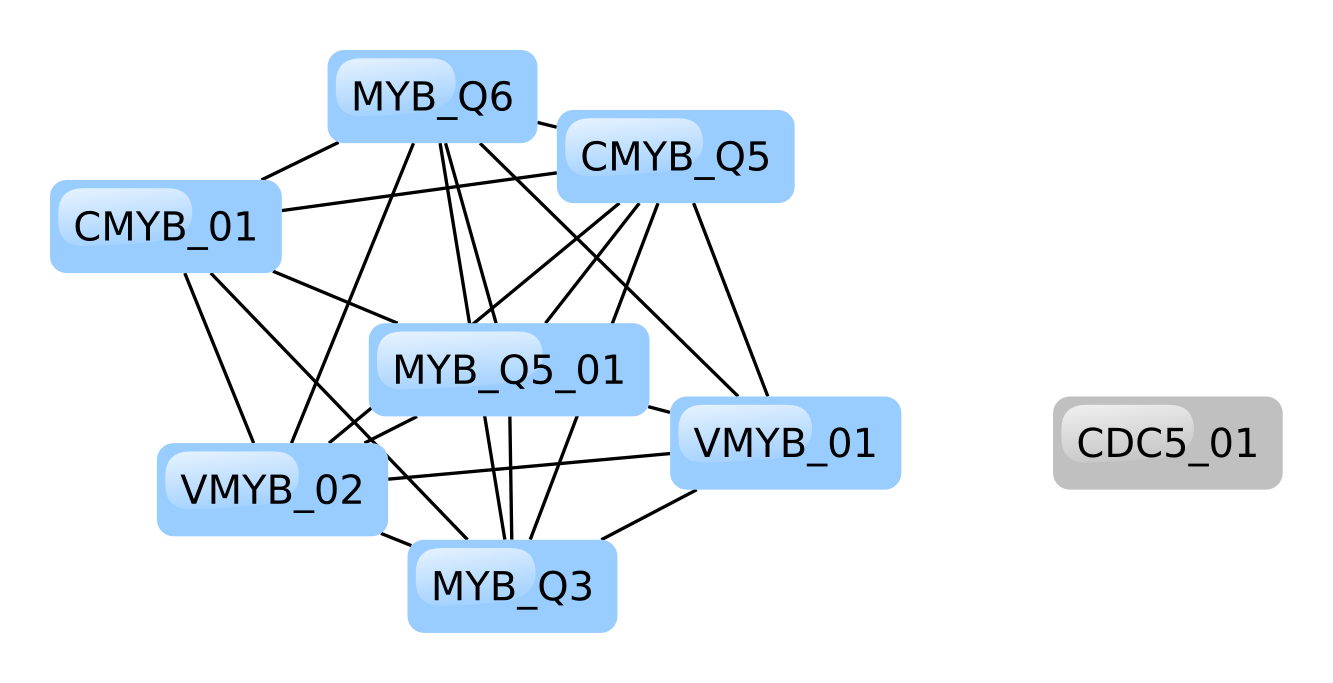 |
| 12 | 7 | **RUNT**  **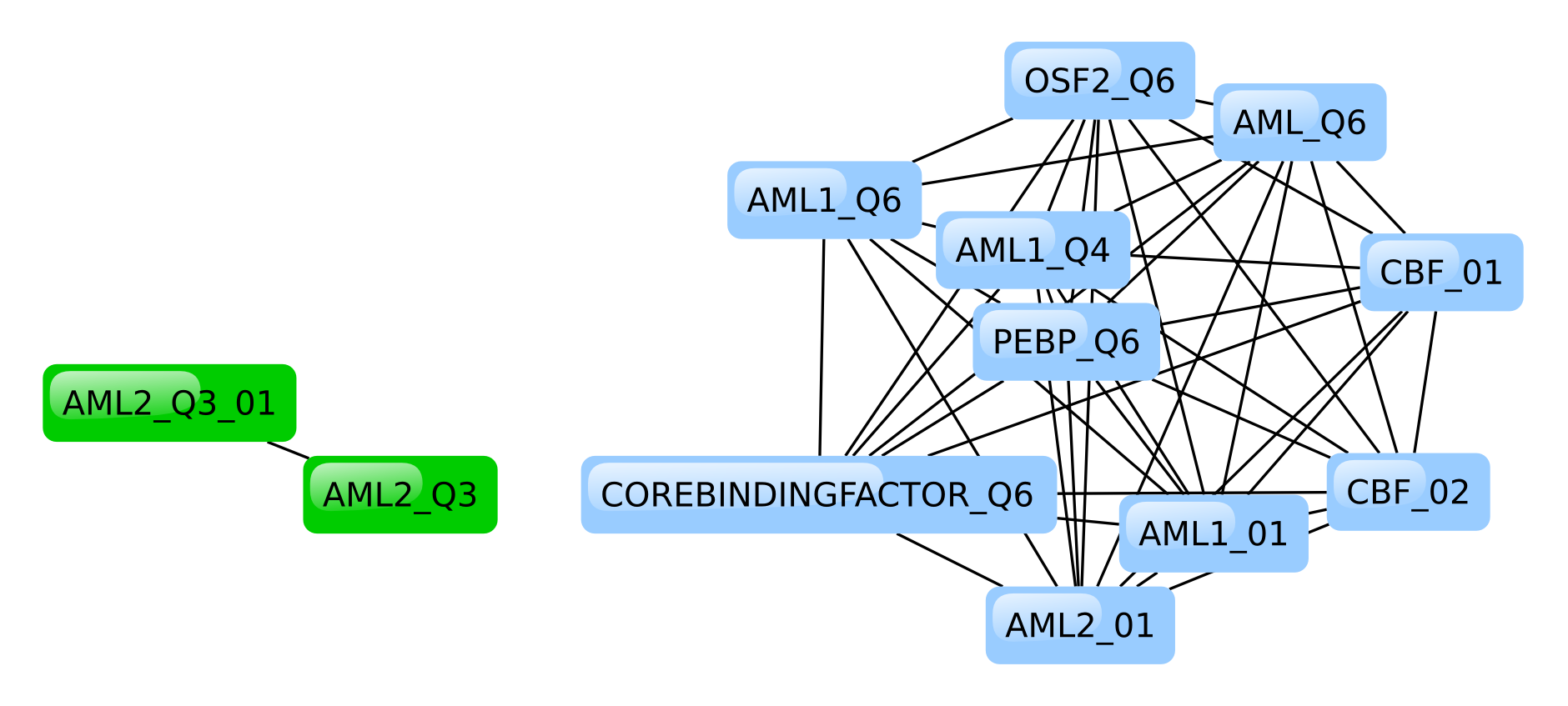** |
| 11 | 8 | **SMAD**  **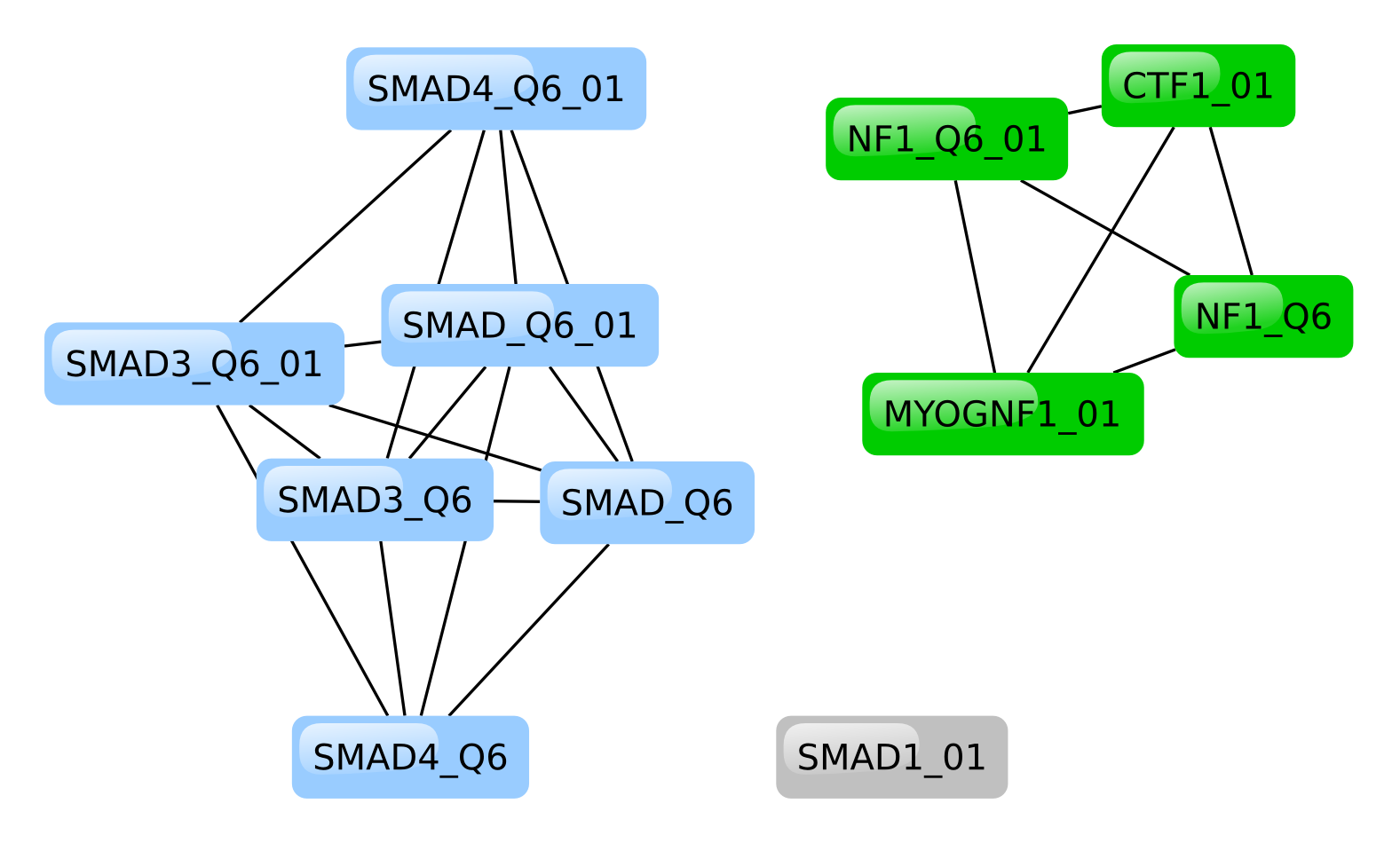** |
| 22 | 9 | **STAT**  **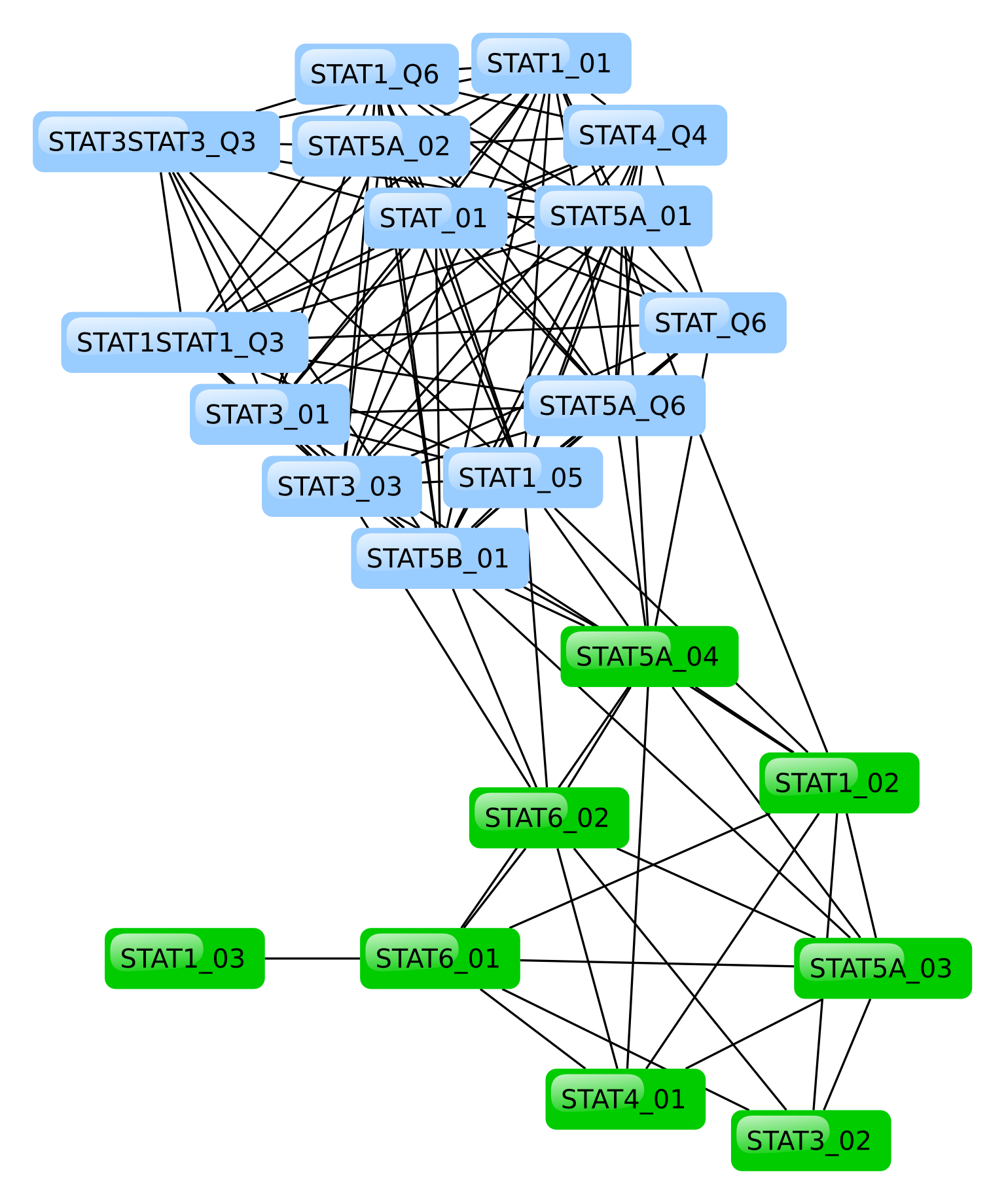** |
| 6 | 10 | **TBP**  **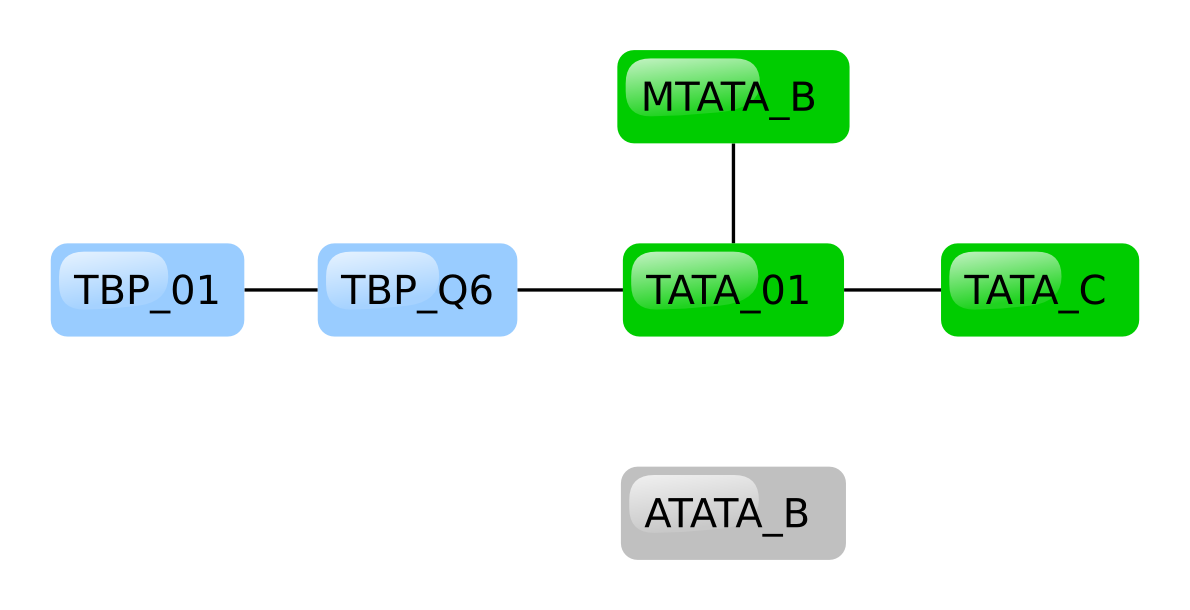** |
