# Supplementary material for: A Discriminative Approach for Unsupervised Clustering of DNA Sequence Motifs
Source: PLoS Comput Biol. 2013 Mar 21;9(3):e1002958. doi: 10.1371/journal.pcbi.1002958 (PMC3605052; doi:10.1371/journal.pcbi.1002958)
Supplement: Text S1 — Motif network clusters and DNA-binding domain phylogenies for the classes BHLH, BZIP, HMG, MADS, REL, SMAD and STAT as well as of clusters obtained for HOX and ZFC2H2 motifs. (DOC) [file pcbi.1002958.s014.doc]

**Text S1**

**Part 1**

Here we describe in detail our results for motif network clusters and corresponding DNA-binding domain phylogenies. Overall, we found compelling agreement between motif and protein groups in the inspected classes. Clusters derived for HMG, SMAD, and MADS matrices (Table S3) were clearly supported by corresponding DNA-binding domain phylogenies as shown by the respective branch coloring (Figure S1A-C). From the perspective of protein similarities the matrices V$SOX_01 and V$SOX9_Q4 (nodes highlighted in Table S3) were falsely assigned to the cluster otherwise made up of LEF/TCF motifs. Also the two STAT motif clusters (Table S3) are in conflict with the protein phylogeny (Figure S1D), because they overlap with respect to annotated binding factors. However, inspection of the clustering produced by m2match disclosed that the STAT class comprised PFMs covering different numbers of half-sites which had been assigned to separate clusters (Figure S1E).


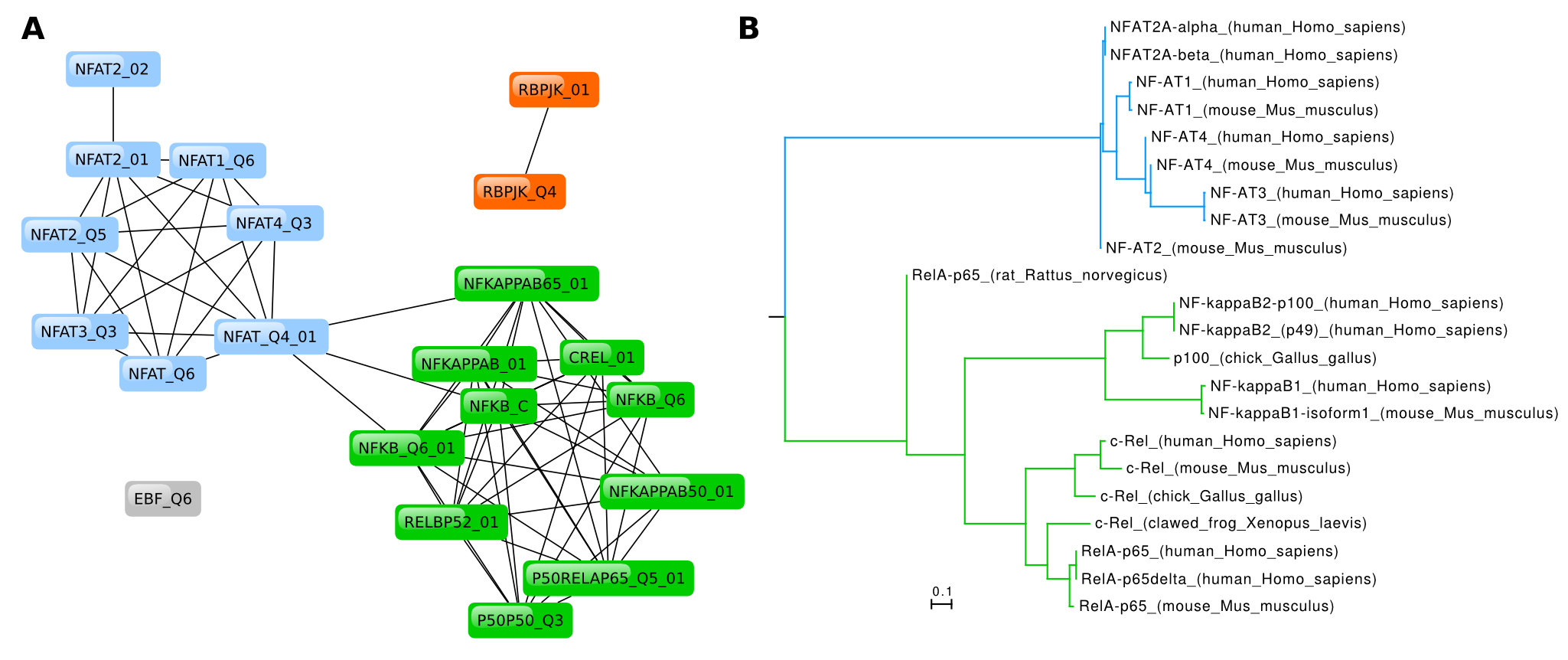
Matrices in the REL class formed a network of three components (Fig. 1). The two smallest components consisted of a single EBF matrix (gray node) and two RBPJK motifs (orange nodes), respectively. In the larger connected component the MCL algorithm identified two clusters. One contained the motifs of the NFAT family (blue nodes) and the other encompassed Rel/NF-kappaB-type motifs (green nodes). These motif clusters match protein subfamilies highlighted in the phylogenetic tree constructed for REL DNA-binding domains (Fig. 2B). We did not consider domains of EBF and RBPJK proteins in the phylogenetic analysis, because their evolutionary relationship to other transcription factors of the REL class is remote and homology was discovered only by detailed analysis of their molecular structures [1,2]. It is therefore plausible that the REL motif network contains no connections between EBF or RBPJK and other REL class PFMs, suggesting that their binding properties have sufficiently diverged to constitute separate motif families.

**Figure 1. Motif network and DNA-binding domain phylogeny for the REL class.** (A) Motif network of REL motifs with colors indicating clusters extracted by MCL. (B) Phylogeny of DNA-binding domains of NFAT and Rel/NF-kappaB-type transcription factors. Branch colors correspond to MCL clusters in A.

**Figure 2. Motif network and DNA-binding domain phylogeny for the BHLH class.** (A) Motif network of BHLH motifs with colors indicating clusters extracted by MCL. (B) Phylogeny of BHLH DNA-binding domains of transcription factors represented by matrices in the motif network. Branch colors correspond to MCL clusters in A. (C) Alignments between matrices from the BHLH-Zip cluster (A, green nodes) and matrices from the SREBP cluster (A, orange nodes). The alignments illustrate the distinct SREBP-specific consensus (top) in comparison to the typical E-box consensus featured by V$MYCMAX_03 and V$SREBP1_01 (middle and bottom).


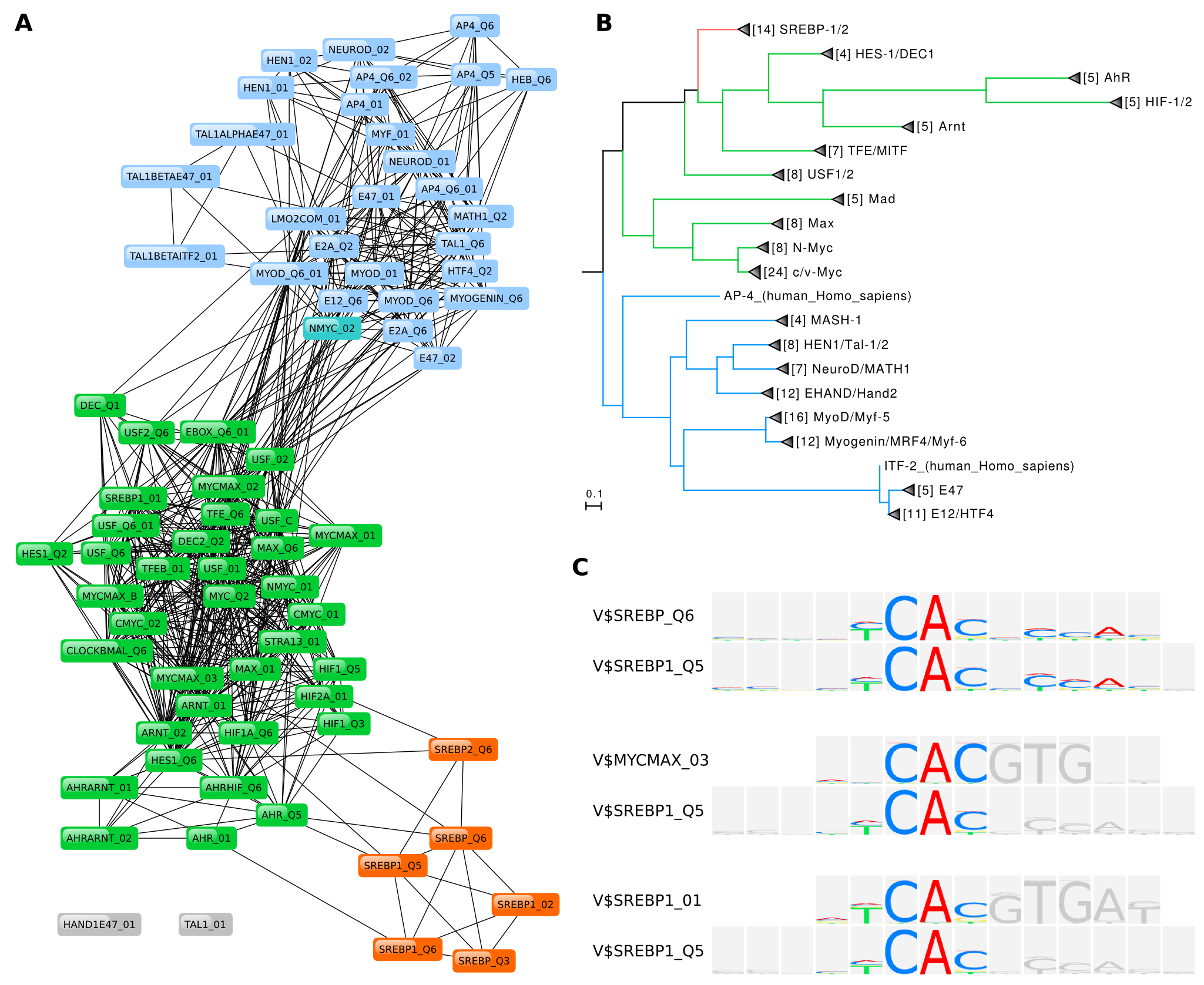
BHLH motifs were assigned to three clusters (Fig. 2A), which can again be recognized as subgroups in the phylogenetic tree constructed for BHLH protein domains (Fig. 2B). The BHLH class comprises two large transcription factor families. In one family the BHLH domain is associated with one or more leucine zipper regions (BHLH-Zip), whereas other proteins of the class do not contain a leucine zipper region (BHLH-only) [3]. The top cluster of the motif network (blue nodes) contained mostly Transfac matrices of BHLH-only factors, whereas matrices of BHLH-Zip factors occupied the other two clusters (green and orange nodes). We consider the placement of the N-Myc (NMYC_02) matrix (turquoise node) within the cluster of BHLH-only PFMs as false positive. Interestingly, the BHLH-Zip factor AP4 was associated with the BHLH-only TFs both in the motif comparison and in the protein domain comparison (which did not consider leucine zipper regions). The cluster of SREBP matrices (orange nodes) warranted further examination. Another SREBP motif (SREBP1_01) was embedded within the large group of BHLH-Zip PFMs (green nodes). According to the protein phylogenetic tree, SREBP factors form a subgroup associated with other domains of the BHLH-Zip type (Fig. 2B). Alignments between matrices of the separate SREBP cluster and a matrix for Myc/Max TFs confirmed that these describe distinct binding site patterns and that the SREBP cluster did not fit the E-box consensus CACGTG (Fig. 2C). The SREBP1_01 PFM however clearly features the E-box pattern and was therefore correctly grouped with respective motifs. The dual specificity of SREBP has previously been described and tested experimentally [4].


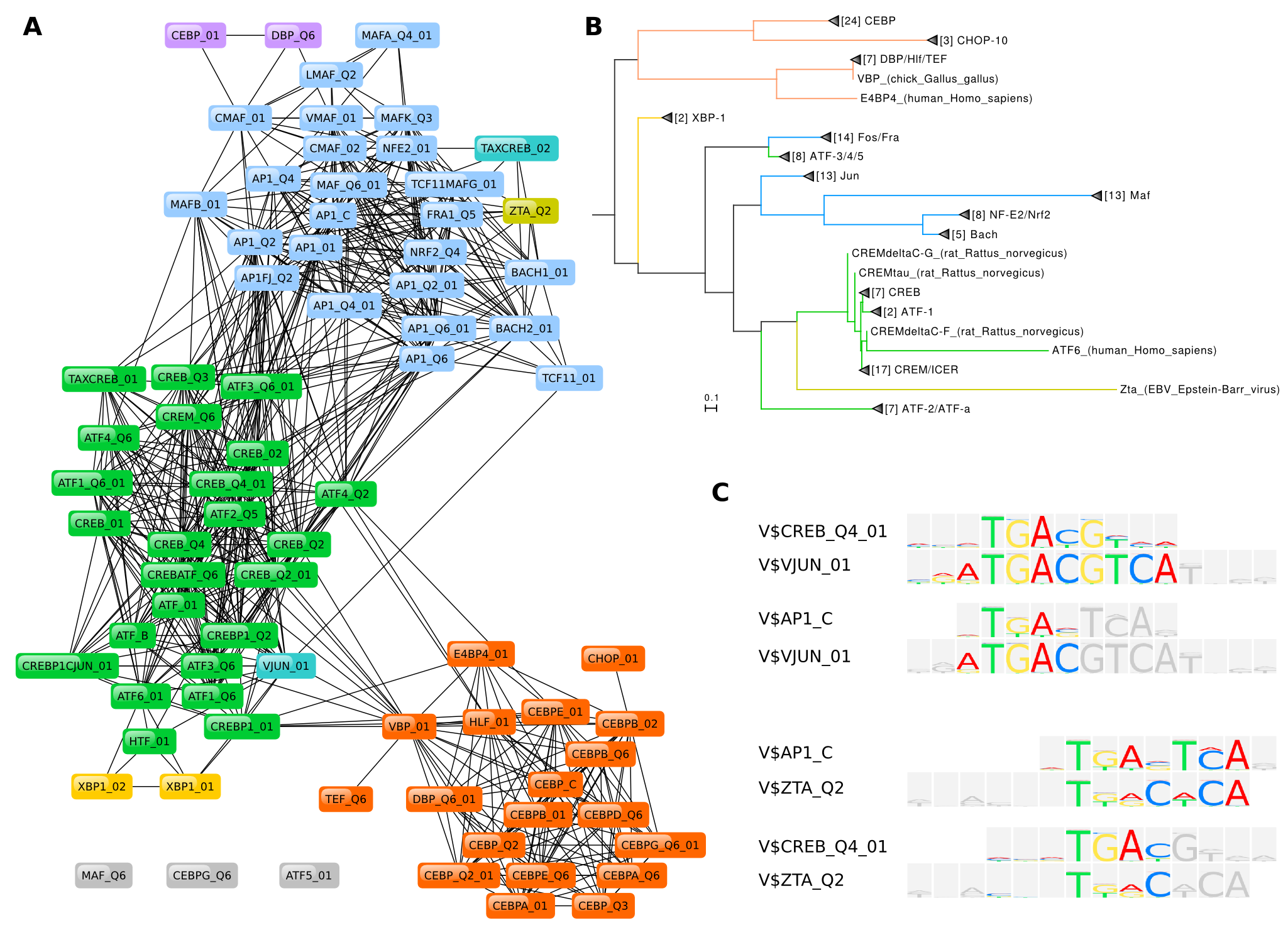
In the BZIP class MCL reported three clusters of motifs corresponding to TF families AP1/MAF (Fig. 3A, blue nodes), CREB/ATF (green nodes), and CEBP/PAR (orange nodes). Some matrices were left as singletons (gray nodes). Also in this class, motif clusters closely correlate with subfamilies of DNA-binding domains as determined by phylogenetic analysis (Fig. 3A and B), albeit there were some exceptions which are discussed in the following.

**Figure 3. Motif network and DNA-binding domain phylogeny for the BZIP class.** (A) Motif network of BZIP motifs with colors indicating clusters extracted by MCL. (B) Phylogeny of BZIP DNA-binding domains of transcription factors represented by matrices in the motif network. Branch colors correspond to MCL clusters in A. (C) Alignments of selected BZIP matrices illustrate that different spacers account for contradictions between motif network locations and protein.

According to relationships on the protein level (Fig. 3B) one would assign the matrices V$TAXCREB_02 and V$ZTA_Q2 to the CREB/ATF cluster, the V$VJUN_01 matrix to the AP1/MAF cluster as well as matrices V$CEBP_01 and V$DBP_Q6 to the CEBP/PAR cluster. Likewise, the protein phylogeny shows a close relationship between the Fos/Fra and ATF3-5 subfamilies, whereas in the motif network all ATF matrices were grouped within the CREB/ATF cluster. We therefore inspected alignments of respective motifs in more detail. V$CEBP_01 and V$DBP_Q6 showed rather weak similarity to connected matrices like V$CMAF_01 (data not shown). Investigation of the other cases revealed that matrices of the CREB/ATF cluster represented binding sites with a CG-consensus spacer between trinucleotide half-sites, whereas in AP1/MAF motifs the half-sites were separated by a single cytosine. In Figure 3C, V$VJUN_01 and V$ZTA_Q2 were aligned to representatives of both clusters. V$CREB_Q4_01 was selected to represent CREB/ATF motifs and V$AP1_C was chosen for AP1/MAF motifs. The visualizations show how alignments were disrupted by the presence of different spacers. Notably, both the v-Jun and the AP1 matrix contain informative TGA and TCA half-sites, which suggests that the placement of v-Jun in the CREB/ATF cluster is due to the additional G-nucleotide within the spacer.

Likewise, alignments of matrices for ATF3, ATF4 and ATF6 with V$CREB_Q4_01 overlap in the first half-site (TGA) and the dinucleotide spacer (CG) which explains why the ATF3 and ATF4 motifs are located within the CREB/ATF group and not with AP1-like (Fos/Fra) motifs as the protein phylogeny suggests (see part S1.2.1 of this document). The opposite was observed for the ZTA matrix (Fig. 3C), which has the *single-C* spacer, but its BZIP domain is more closely related to proteins of the CREB/ATF group (Figure 3B). We also inspected the XBP1 matrices (V$XBP1_01 and V$XBP1_02), which were assigned to the CREB/ATF cluster (Fig. 3A, yellow nodes). Again, we found that these motifs contain the half-site TGA (where the 5'-T is missing in the V$XBP1_02 matrix) followed by the CG-spacer. XBP1 shares with ATF6 the partial TGACGTG consensus (S1.2.2 below).

We think that the V$TAXCREB_02 matrix was falsely connected to the AP1/MAF motif cluster (Fig. 3A). Its pattern shows the CG-spacer and exhibits high similarity to V$TAXCREB_01 with the exception of GC-rich regions that are located on opposite sides of the two PFMs. (S1.2.3). We tested whether consideration of information coverage may have caused the false assignment of V$TAXCREB_02, but found the same arrangement when the BZIP motif network was constructed using ED instead of ED.sqr scores (data not shown).

Finally, MCL predicted 12 motif clusters in the HOX class as well as ZFC2H2 class with different numbers of singleton PFMs (Table S1). HOX and ZFC2H2 were the largest classes in our study set. The result for the HOX network is visualized in Figure S2 and motif clusters are listed in Text S2. The second largest group extracted by the MCL algorithm was mainly composed of POU-type motifs (Text S2, line 2). Furthermore, we obtained clusters exclusively or mostly comprising matrices for NKX-, PBX-, IRX-, CDX-, or PAX-type transcription factors. The clustering of PAX PFMs is particularly interesting as we allotted these matrices, including those for PAX-only factors, in the class of HOX motifs in order to determine whether these would associate with other HOX motifs or occupy a separate group. Hence, our result suggests that PAX factors comprise a group with distinct binding properties from other HOX proteins. Other PAX matrices were assigned to the largest cluster (Text S2, line 1). Based on the ZFC2H2 motif network MCL revealed 12 groups and 18 matrices were disconnected (Text S3 and Figure S3). The largest group contained matrices of SP1, SP3, EGR as well as GLI transcription factors. Inspection of the smaller clusters showed that these connected matrices of closely related proteins (Figure S3).

**References**

1. Kovall RA, Hendrickson WA (2004) Crystal structure of the nuclear effector of Notch signaling, CSL, bound to DNA. EMBO J 23: 3441-3451.

2. Treiber N, Treiber T, Zocher G, Grosschedl R (2010) Structure of an Ebf1:DNA complex reveals unusual DNA recognition and structural homology with Rel proteins. Genes Dev 24:2270-2275.

3. Wingender E (1997) Classification of eukaryotic transcription factors. Mol Biol (Mosk) 31:584-600.

4. Kim JB, Spotts GD, Halvorsen YD, Shih HM, Ellenberger T, et al. (1995) Dual DNA binding specificity of ADD1/SREBP1 controlled by a single amino acid in the basic helix-loop-helix domain. Mol Cell Biol 15: 2582-2588.

**Part 2**

**S1.2.1**

**Alignments with ATF matrices**

**
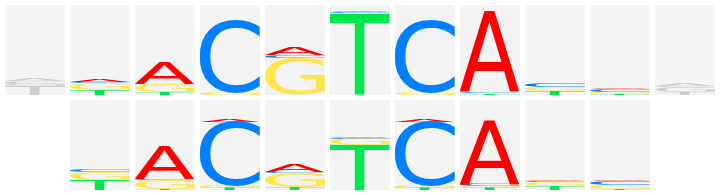
**

V$CREB_Q4_01

V$ATF4_Q6

V$CREB_Q4_01


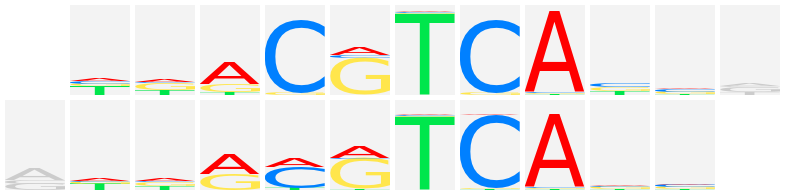
V$ATF3_Q6_02

V$CREB_Q4_01


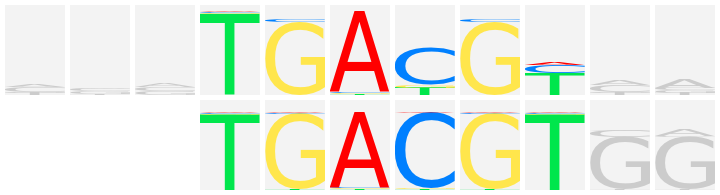
V$ATF6_01

**S1.2.2**

**Alignments with XBP1 matrices**

V$XBP1_02


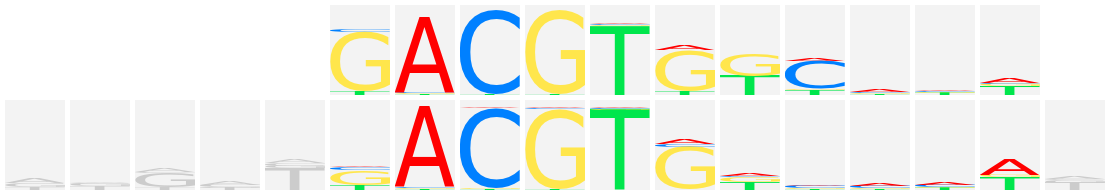
V$XBP1_01

V$ATF6_01


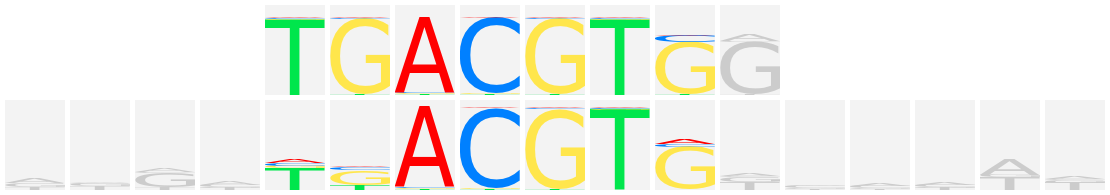
V$XBP1_01

V$ATF6_01


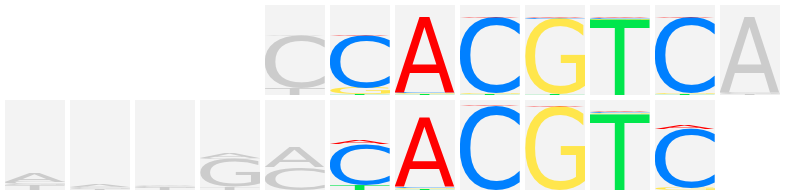
V$XBP1_02

V$CREB_Q4_01


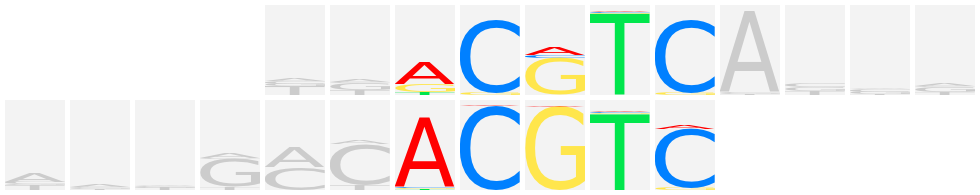
V$XBP1_02

**S1.2.3**

**Alignments with TAXCREB matrices**

**V$TAXCREB_02**

V$AP1_C


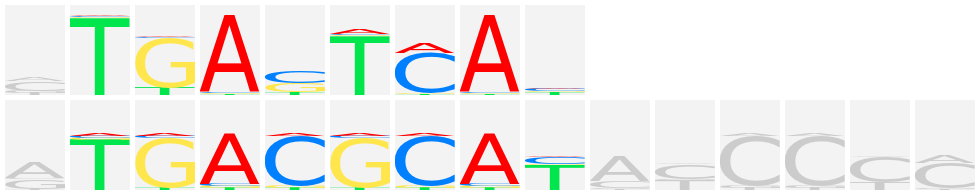


V$TAXCREB_02


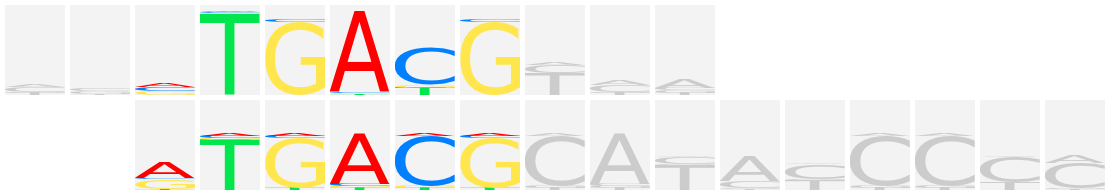
V$CREB_Q4_01

V$TAXCREB_02

**V$TAXCREB_01 vs. V$TAXCREB_02**

V$TAXCREB_01


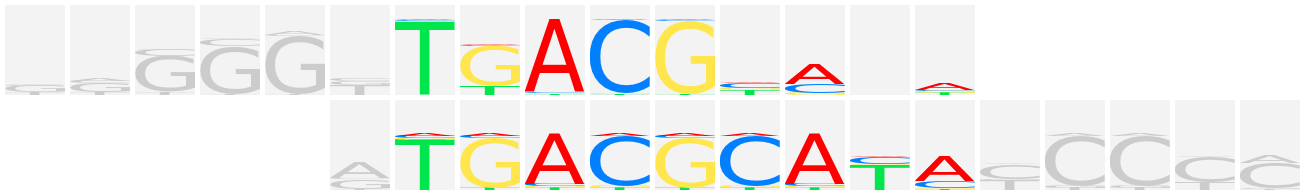
V$TAXCREB_02

**V$TAXCREB_01**


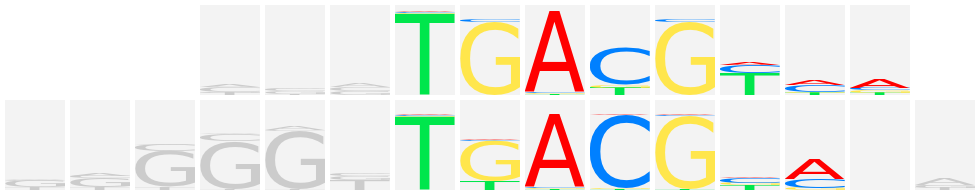
V$CREB_Q4_01

V$TAXCREB_01

V$AP1_C


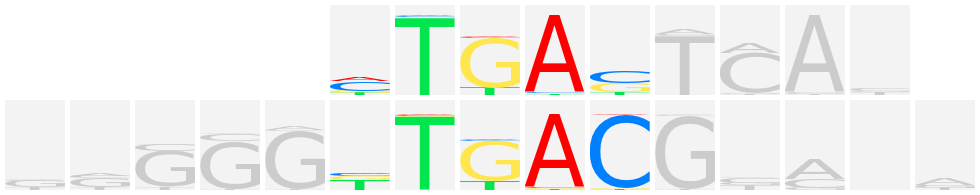


V$TAXCREB_01
